# Downregulated long noncoding RNA ALDBGALG0000005049 induces inflammation in chicken muscle suffered from selenium deficiency by regulating stearoyl-CoA desaturase

**DOI:** 10.18632/oncotarget.17187

**Published:** 2017-04-18

**Authors:** Ruifeng Fan, Changyu Cao, Xia Zhao, Qunxiang Shi, Jinxin Zhao, Shiwen Xu

**Affiliations:** ^1^ College of Veterinary Medicine, Northeast Agricultural University, Harbin 150030, P. R. China

**Keywords:** lncRNA, inflammation, Se deficiency, chicken muscle, stearoyl-CoA desaturase

## Abstract

Long non-coding RNAs (lncRNAs) have been demonstrated to play a pivotal role in proliferation and differentiation of muscles. However, the study on the roles of lncRNAs in Selenium (Se) deficiency induced muscle injury is still unclear. In this study, deep sequencing was performed to profile lncRNAs and mRNAs of the muscles from the Se deficiency (-Se group) and control (C group) chickens. The results revealed that 38 lncRNAs (23 up-regulated and 15 down-regulated) and 687 mRNAs (285 up-regulated and 402 down-regulated) were significantly dysregulated expressed, and the significantly dysregulated pathway including Phagosome, Cardiac muscle contraction, and Peroxisome Proliferator-Activated Receptor (PPAR) in -Se group. The regulatory relationship between ALDBGALG0000005049 and stearoyl-CoA desaturase (SCD), which involved in PPAR pathway was verified. The results also showed that the decreased expressions of SCD, PPARα, PPARβ and PPARγ, and the increased expressions of interleukin (IL)-1β, IL-6, IL-8, and chemokine (C-C motif) ligand 4 (CCL4) along with silencing of ALDBGALG0000005049 in chicken myoblasts. Moreover, increased expressions of IL-1β, IL-6, IL-8, and CCL4 and inflammatory cell infiltration in microstructure of chicken muscles treated with Se deficiency were observed. This study displayed an overview of aberrantly expressed lncRNAs and mRNAs profiles and PPAR pathway, and revealed that downregulation of ALDBGALG0000005049 caused inflammation by regulating SCD in chicken muscle resulted from Se deficiency.

## INTRODUCTION

Se, as an essential micronutrient, plays an important role in various biological activities and Se deficiency induces some diseases including exudative diathesis [1], pancreatic atrophy [2], and nutritional muscular dystrophy [3] in chickens. Recent studies have shown that dysregulated genes expression in selenoproteins [4], inflammation [5], apoptosis, and calcium signaling [6], which might contribute to Se deficiency-mediated nutritional muscular dystrophy. However, the concrete molecular mechanism of Se deficiency-related muscle injuries remains unclear.

LncRNAs, as a new class of transcripts (longer than 200 bases) without evident protein-coding capacity, have been recently identified to be ubiquitous transcribed in eukaryotic genome [7]. Accumulating evidences demonstrated that lncRNAs regulated genes expression by influencing the transcription, translation, and chromatin modification during genetic processes [8–10]. In particular, increasing studies suggested that aberrant expressions of lncRNAs could be implicated in many diseases such as cancer [11], neurological [12], and cardiovascular disease [13] in humans and Marek's disease [14] in chickens. Recent studies also indicated that certain lncRNAs exerted their regulatory function in muscles development and diseases. For example, long intergenic noncoding RNA, which is associated with muscle differentiation (linc-MD1) is greatly decreased in the muscles of Duchenne Muscular Dystrophy (DMD) patients, and overexpression of linc-MD1 can restore the defective myogenic differentiation [15]. Whereas long noncoding RNA 31 expressed in several tissues including skeletal muscles, inhibits myogenic differentiation [16].

Skeletal muscle acts as the primary location of glycogen storage, insulin-mediated glucose utilization, lipid metabolism, and fatty acid oxidation [17, 18]. PPAR signaling pathway, which is well recognized in interfering metabolic disorders and in muscle adaptation to physical exercise and fasting, plays a chief role in muscle proliferation and differentiation [19–21]. Particularly, the PPARβ/δ is also closely associated with skeletal muscle metabolism, plasticity, and disorders [22]. Meanwhile, the expression of some genes including SCD regulated by PPARβ/δ. SCD, as an endoplasmic reticulum iron-containing microsomal enzyme, converts saturated fatty acids (SFA) into monounsaturated fatty acids and is necessary for skeletal muscle lipid metabolism. It is noted that upregulated SCD1 can protect the skeletal muscle cells, which exposed to palmitic acid from lipotoxicity and insulin resistance by reducing the expressions of the factors implicating in inflammation (IL-6 and IL-8), and endoplasmic reticulum stress [23]. Similarly, prior immunologic studies also indicated that SCD1-deficient mice has skin that characterized by chronic dermal inflammation [24, 25], and has atherosclerosis possessed the characteristic of inflammation [26]. Se deficiency induced inflammation in chicken muscles, aorta vessels, and kidneys by excessing production of the inflammatory factors have been revealed [5, 27, 28]. These results showed that the productions of inflammatory factors were increased following Se-deficiency and indicated that factors or pathways that can induce the inflammatory response should be paid more attention in tissues and organs damages induced by Se deficiency. However, the studies about the effects of some specific lncRNAs and SCD on the inflammation in chicken muscle treated with Se deficiency are scarce.

In the current study, to explore roles of lncRNAs and their target genes in the muscle injury induced by Se deficiency, the deep sequencing was performed and dysregulated expressed lncRNAs and mRNAs and the PPAR pathway were revealed in muscles suffered from Se deficiency. Meanwhile, the regulatory relationship between ALDBGALG0000005049 and SCD was verified and downregulated ALDBGALG0000005049 induced inflammation by targeting SCD was revealed. We suggested that ALDBGALG0000005049-SCD might contribute to development of muscle damage induced by Se deficiency and modulation of ALDBGALG0000005049-SCD could become a novel strategy for muscle damage induced by Se deficiency.

## RESULTS

### Se deficiency induced inflammation in chicken muscles

**Figure 1:**
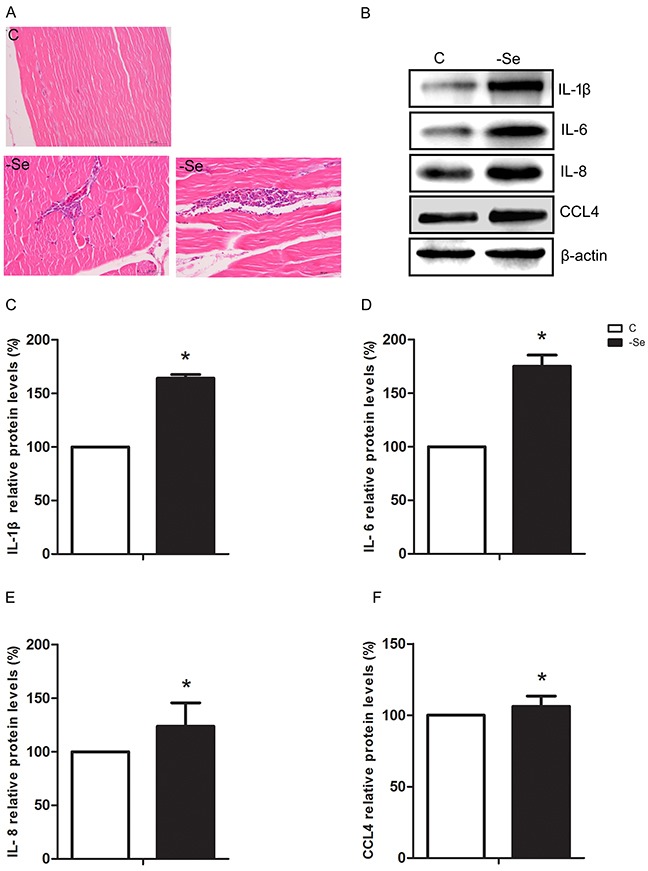
Se deficiency caused inflammation response in chicken muscles **(A)** Photomicrograph of C group and -Se group muscles, ×400. Upper panel represented the photomicrographs of C group muscle, and lower panels represented the photomicrographs of -Se group muscle. **(B)** The protein expressions of IL-1β, IL-6, IL-8, and CCL4 in the C group and -Se group muscles. **(C-F)** Data presented the relative expressions of IL-1β, IL-6, IL-8, and CCL4 protein levels (standardized to the intensity of β-actin). The data are expressed as the means ± SD.* Significant difference from the corresponding control (P<0.05).

We found that the histopathological structure of muscles in C group was normal and intact, however, the muscles were represented inflammatory cell infiltration in -Se group (Figure 1A). Moreover, we further explored the expression levels of IL-1β, IL-6, IL-8, and CCL4 involved in inflammation induced by Se deficiency, and the results showed that protein expressions of IL-1β, IL-6, IL-8, and CCL4 were significantly increased (p<0.05) induced by Se deficiency (Figure 1B-1F). Thus Se deficiency induced inflammation damage in chicken muscles.

### Overview of lncRNAs and mRNAs expression profiles in muscles

**Figure 2:**
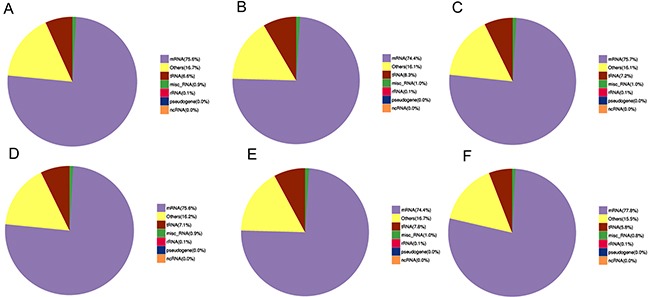
Classification of mapped reads The classification of mapped reads in samples CM1 **(A)**, CM2 **(B)**, CM3 **(C)**, -SeM1 **(D)**, -SeM2 **(E)** and -SeM3 **(F)**.

**Figure 3:**
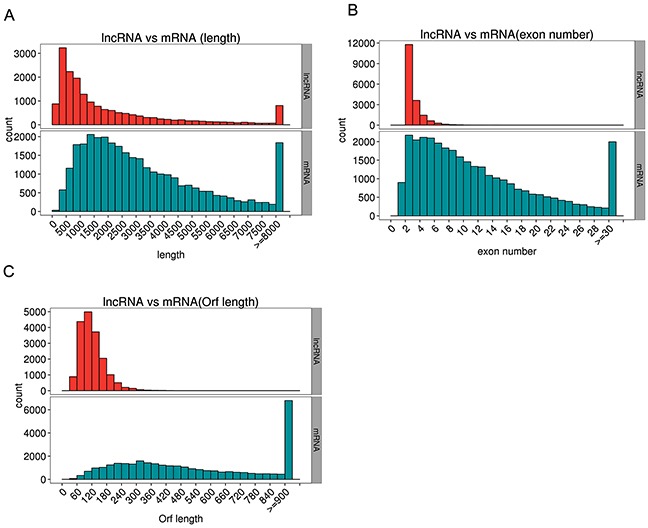
The genomic features of lncRNAs The candidate lncRNAs identified were shorter in transcript length **(A)**, fewer exons **(B)** and shorter in orf length than mRNAs **(C)**.

We explored the deep sequencing to determine the expression profiles of lncRNAs and mRNAs in the muscles of C group (CM1, CM2, and CM3) and that of -Se group (-SeM1, -SeM2, and -SeM3). According to the methods of library preparation, corresponding libraries were established and the data output quality was shown in Table 1. Table 2 show that the result of reads compared with the reference genome, and Figure 2 shows that the classification of mapped reads. Finally, a total of 18,060 lncRNAs and 35,095 mRNAs were detected. The novel mRNAs were considered by any of the four tools (CNCI, CPC, Pfam-scan, and PhyloCSF) and 2,969 novel mRNAs were detected in this study. The results also showed that 687 mRNAs and 38 lncRNAs were differentially expressed in -Se group compared with C group. Among them, 285 mRNAs were upregulated and 402 mRNAs were downregulated in -Se group. Regarding the lncRNAs, we found that 23 were upregulated and 15 were downregulated in -Se group. All the lncRNAs and mRNAs that were differently expressed with q <0.05 are listed in Supplementary Table 1. In addition, we also observed the genomic features of lncRNAs, the results showed that the candidate lncRNAs identified were shorter in transcript length (Figure 3A) fewer exons (Figure 3B) and shorter in orf length than mRNAs (Figure 3C).

**Table 1:** The data output quality of lncRNAs and mRNAs profiles in this study

Sample name	Raw reads	Clean reads	Error rate(%)	Q20(%)	Q30(%)	GC content(%)
CM1	43401487.00	41505023.00	0.03	96.65	93.23	48.68
CM2	50209586.00	49201700.00	0.03	96.44	92.79	48.89
CM3	52231730.00	50685786.00	0.03	96.60	93.17	48.47
-SeM1	49337439.00	47268388.00	0.03	96.67	93.23	48.80
-SeM2	51470835.00	49293539.00	0.03	96.70	93.31	48.84
-SeM3	49505032.00	47560567.00	0.03	96.73	93.36	49.25

**Table 2:** The reads compared with the reference genome

Sample name	CM1	CM2	CM3	-SeM1	-SeM2	-SeM3
Total reads	83010046.00	98403400.00	101371572.00	94536776.00	98587078.00	95121134.00
Total mapped	72626663 (87.49%)	85813152 (87.21%)	88743743 (87.54%)	82600375 (87.37%)	85140309 (86.36%)	82062723 (86.27%)
Multiple mapped	8606169 (10.37%)	9493163 (9.65%)	9772963 (9.64%)	9902005 (10.47%)	10650590 (10.8%)	9827915 (10.33%)
Uniquely mapped	64020494 (77.12%)	76319989 (77.56%)	78970780 (77.9%)	72698370 (76.9%)	74489719 (75.56%)	72234808 (75.94%)
Non-splice reads	41902024 (50.48%)	50779499 (51.6%)	51737671 (51.04%)	46733889 (49.43%)	49858071 (50.57%)	45244393 (47.57%)
Splice reads	22118470 (26.65%)	25540490 (25.95%)	27233109 (26.86%)	25964481 (27.46%)	24631648 (24.98%)	26990415 (28.37%)

### Functional enrichment analysis

**Figure 4:**
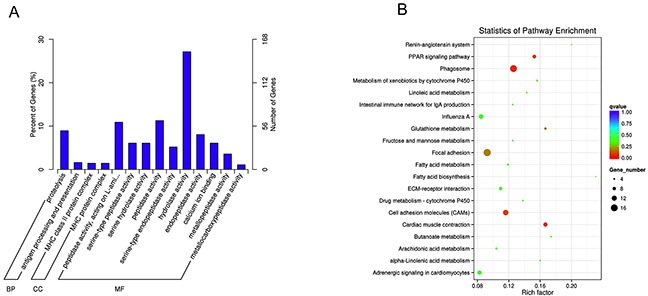
GO and KEGG pathway analyses of differentially expressed mRNAs **(A)** GO analysis of differentially expressed mRNAs. **(B)** KEGG Pathway analysis of differentially expressed mRNAs.

In order to identify the function of coding transcripts, the Gene ontology (GO) analysis for all of the differentially expressed mRNAs was performed. The results showed that the genes corresponding to the differentially expressed mRNA transcripts were involved in 943, 219 and 554 GO terms in the biological process network, cellular component network, and molecular function network, respectively. Interestingly, the results showed that the differentially expressed mRNAs related to molecular function network were specific to peptidase activity (GO: 0070011), acting on L-amino acid peptides (GO: 0070011), serine-type peptidase activity (GO: 0008236), serine hydrolase activity (GO: 0017171), and serine-type endopeptidase activity (GO: 0004252). The top 14 significant GO terms associated with dysregulated mRNAs are shown in Figure 4A. To make a further understanding between the differentially expressed mRNAs and pathways, the Kyoto Encyclopedia of Genes and Genomes (KEGG) pathway analysis was also explored to analyze the differentially expressed mRNAs. The results showed that a total of 108 pathways were enriched among the differentially expressed mRNAs and 12 pathways were significantly enriched (p<0.05) including Phagosome, Cardiac muscle contraction, PPAR signaling pathway, Cell adhesion molecules (CAMs), Focal adhesion, Glutathione metabolism, Metabolism of xenobiotics by cytochrome P450, ECM-receptor interaction, Butanoate metabolism, Fatty acid biosynthesis, Alpha-Linolenic acid metabolism, and Renin-angiotensin system. The top 20 enriched pathways of differentially expressed mRNAs are shown in Figure 4B.

### Target gene prediction

In order to verify the functions of lncRNAs, the potential targets of lncRNAs were predicted in cis using 100kb as the cutoff. The prediction identified 33 lncRNA-mRNA pairs included 33 lncRNAs and 178 mRNAs (Supplementary Table 2).

### Construction of the lncRNA-mRNA co-expression network

**Figure 5:**
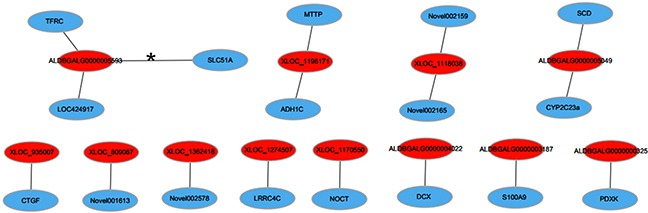
LncRNA-mRNA co-expression network Red nodes represent lncRNAs, blue nodes represent mRNAs. The line, marked asterisk, between lncRNAs and mRNAs indicate a negative correlation, while other lines indicate a positive correlation.

The lncRNA-mRNA co-expression networks were built based on the results of correlation analyses of the differentially expressed lncRNAs and mRNAs. To further optimize the network, we selected the mRNAs, which were the target genes of corresponding lncRNAs in cis. Finally, 12 co-expression networks were constructed, which included 12 lncRNAs and 17 mRNAs (Figure 5). Hereinto, only one lncRNA-mRNA gene pair (ALDBGALG0000005593-solute carrier family 51 alpha subunit) was regulated in the opposite direction. It is noteworthy that the lncRNA-mRNA gene pair (ALDBGALG0000005049-SCD) involving in the PPAR signaling pathway, which was a pathway significantly enriched by KEGG pathway analyzing.

### Real-time quantitative PCR (qPCR) validation

**Figure 6:**
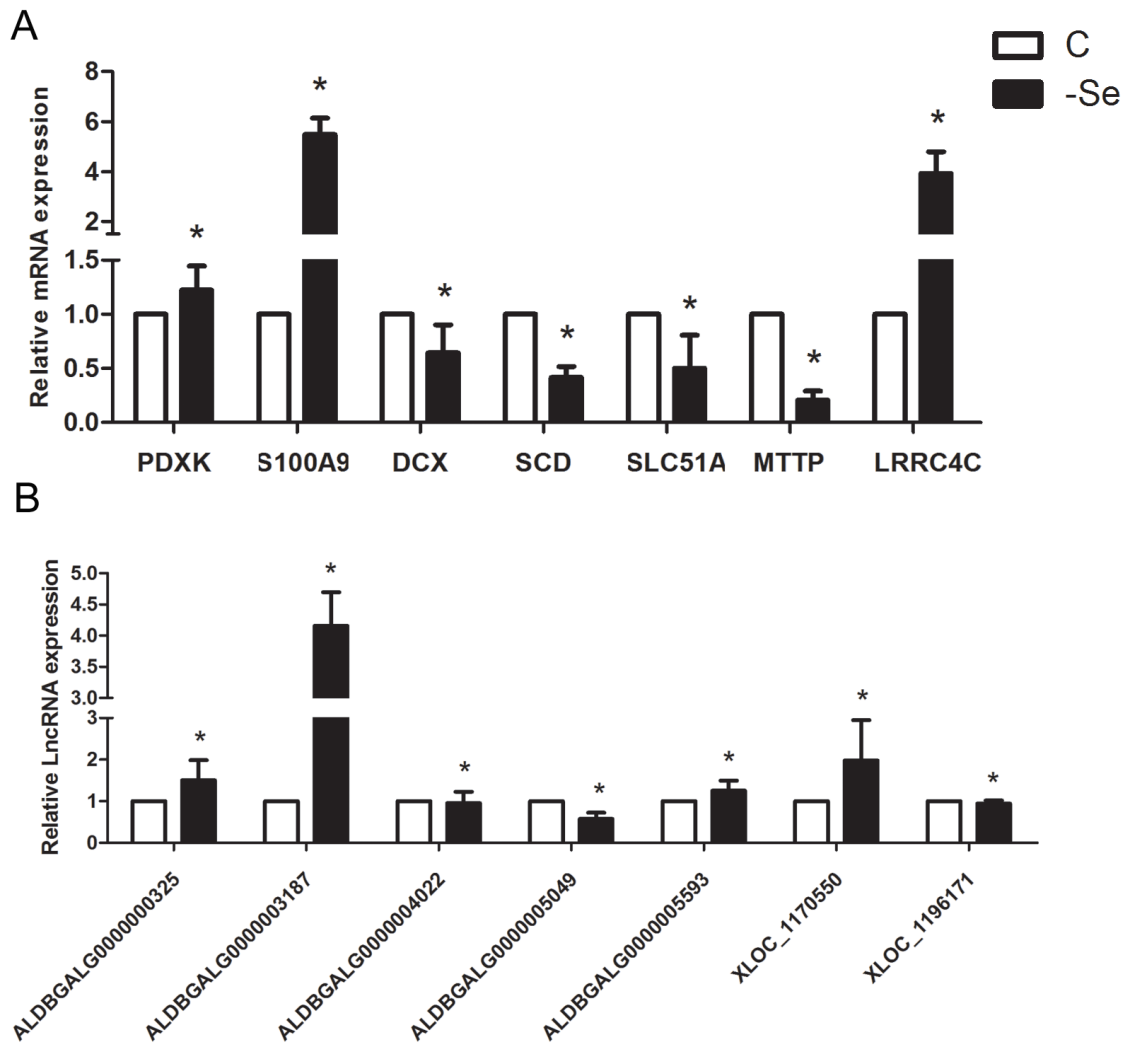
QPCR validation of the profiles lncRNAs and mRNAs **(A)** The expression levels of mRNAs. **(B)** The expression levels of lncRNAs. The data are expressed as the means ± SD.* Significant difference from the corresponding control (P<0.05).

In order to verify the reliability of the deep sequencing results, qPCR was explored to measure the expression levels of 7 lncRNAs and 7 mRNAs selected randomly from the lncRNA-mRNA co-expression network. As shown in Figure 6, the expression levels of ALDBGALG0000000325, ALDBGALG0000003187, ALDBGALG0000004022, ALDBGALG0000005593, XLOC_1170550, PDXK, S100A9, and LRRC4C were significantly increased (p<0.05), and the expression levels of ALDBGALG0000005049, XLOC_1196171, DCX, SCD, SLC51A, and MTTP were significantly decreased (p<0.05) in -Se group compared to that in C group (p<0.05). Thus, the results of qPCR were consistent with the expression trends of the deep sequencing.

### Silencing lncRNA-ALDBGALG0000005049 induced inflammation in chicken myoblasts

**Figure 7:**
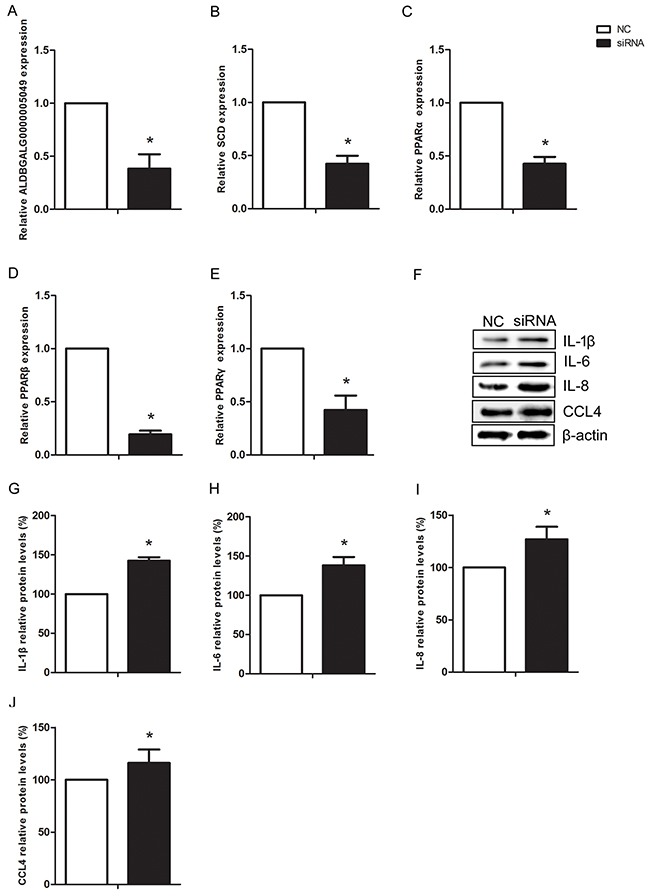
Silencing lncRNA-ALDBGALG0000005049 induced inflammation response in chicken myoblasts **(A-E)** Relative ALDBGALG0000005049, SCD, PPARα, PPARβ, and PPARγ expression after ALDBGALG0000005049 silencing in chicken myoblasts. **(F)** The protein expressions of IL-1β, IL-6, IL-8, and CCL4 in the NC group and siRNA group chicken myoblasts. **(G-J)** Data presented the relative expressions of IL-1β, IL-6, IL-8, and CCL4 protein levels (standardized to the intensity of β-actin). The data are expressed as the means ± SD.* Significant difference from the corresponding control (P<0.05).

To further confirm the regulatory relationship between ALDBGALG0000005049 and SCD, we silenced the expression of lncRNA-ALDBGALG0000005049 using small interfering RNA (siRNA) in chicken myoblasts. Firstly, we observed the silencing efficiency of the designed siRNA. As shown in Figure 7A, the expression of ALDBGALG0000005049 was significantly decreased by 62% (p<0.05) after 48h transaction of siRNA. The result indicated that the designed siRNA for ALDBGALG0000005049 was effective in chicken myoblasts. The results also showed that the mRNA expression levels of SCD, PPARα, PPARβ/δ, and PPARγ were significantly decreased (p<0.05) after transfected with ALDBGALG0000005049-siRNA in chicken myoblasts (Figure 7B–7E). To explore whether the decreased expression SCD due to silencing of ALDBGALG0000005049 can affect the factors involving in inflammation, the expressions of IL-1β, IL-6, IL-8, and CCL4 were checked in chicken myoblasts. As shown in Figure 7F–7J, silencing of ALDBGALG0000005049 significantly increased (p<0.05) the protein expression levels of IL-1β, IL-6, IL-8, and CCL4 in chicken myoblasts. Therefore, silencing ALDBGALG0000005049 induced inflammation in chicken myoblasts.

## DISCUSSION

LncRNAs have been demonstrated to play key roles in different biological processes and were increasingly recognized as contributors to the pathology of some diseases. In chicken, lncRNAs were recently found to play an important role in chicken viral infection diseases [29]. It has been reported that significantly increases in lncRNA (MYOZ1, TMEM130 and UBE2QL1) abundance in bursal disease virus-stimulated dendritic cells [30]. However, the study on the roles of lncRNAs in muscle damage, particularly in Se deficiency induces nutritional muscular dystrophy is still unclear. In this study, we used the deep sequencing to detect the profiles of lncRNAs and mRNAs in the muscles between C group and -Se group. The results revealed that the following novel findings: (1) Aberrant lncRNA and mRNA profiles and dysregulated PPAR pathway in the muscles treated with Se deficiency. (2) Certified the regulatory relationship between ALDBGALG0000005049 and SCD. (3) Downregulated ALDBGALG0000005049 induced inflammation by regulating SCD in chicken muscles resulted from Se deficiency. To our knowledge, this is the first study that presented the profiles of lncRNAs and mRNAs in the Se-deficient muscles, and it offered helpful information to understand the roles of lncRNA ALDBGALG0000005049-SCD in pathogenic mechanism of muscle damage induced by Se deficiency.

In this study, we found 26,401 known mRNAs, 8,694 novel mRNAs, 18,060 candidate lncRNAs, and differently expression of 38 lncRNAs and 687 mRNAs. Compared to the previous report on the lncRNAs profile in muscle development during embryo [31], this study provided more information to the lncRNAs profile on the muscle damage in chicken. To further reveal the pathogenesis of Se deficiency mediate muscle injury, the differentially expressed mRNAs were disposed by GO analysis and KEGG pathway analysis. The results of GO analysis showed that the differentially expressed mRNAs related to molecular function network were specific to disorder of metabolism. The results of KEGG pathway analysis showed that 12 pathways were significantly enriched. Notably, the PPAR signaling pathway (gga03320) caught our attention because this pathway involved in skeletal muscle metabolism. PPARs which comprise some members including PPARα, PPARβ/δ, and PPARγ, belong to the nuclear receptor superfamily of transcription factors [32], and PPARβ/δ has emerged as a key transcription factor in skeletal muscle [33]. Some prior studies have demonstrated that GW501516 increased the catabolism of fatty acid, cholesterol efflux, and energy expenditure in muscle by activating PPARβ/δ [34, 35]. Prior studies by S. Luquet, et al., [36] and Y.X. Wang, et al., [37] indicated that muscle-specific PPARβ/δ played important roles in muscle remodeling, through overexpression or expression actived by VP16-PPARβ/δ protein in transgenic mice. In addition, other studies have revealed that activation of PPARβ/δ improved the DMD phenotype [38], and promoted calcineurin-dependent fiber remodeling [39]. Notably, expressions of the genes including SCD involved in skeletal muscle metabolism were influenced by the presence and absence of the PPARβ/δ agonists [34]. In this study, we established the lncRNA-mRNA co-expression network and the lncRNA-mRNA gene pair (ALDBGALG0000005049-SCD) aroused our interest because the SCD was a participator of PPAR pathway, which was a pathway significantly enriched by KEGG pathway analyzing the differently expressed mRNA.

In order to deep research the regulatory relationship between ALDBGALG0000005049 and SCD, we designed the special siRNA for SCD and transfected it to the chicken myoblasts. The results showed that knocking down ALDBGALG0000005049 significantly decreased the mRNA expression of SCD. Combined with the lncRNA-mRNA co-expression network, it is reasonable to hypothesize that the regulation relationship between ALDBGALG0000005049 and SCD. In the lipid metabolism of skeletal muscle or muscle cells, SFA can cause cellular stress and inflammation through several different pathways [40–42]. Thus as a restrict enzyme of converting SFA into monounsaturated fatty acids, SCD has been gained comprehensive attention. Previous studies indicated that overexpression of SCD1 prevented the inflammatory response to palmitate exposure in human myotubes [43], and SCD1-deficient mice have increased inflammation [44]. Thus these researches promoted us to explore whether silencing ALDBGALG0000005049 mediated downregulation of SCD induces the inflammation in chicken myoblasts. The result showed that expressions of IL-1β, IL-6, IL-8, and CCL4 were increased. Aberrantly PPAR pathway has been revealed from the results of KEGG analysis *in vivo*. It was worth noting that after silencing of ALDBGALG0000005049 in chicken myoblasts, the expressions of PPARα, PPARβ, and PPARγ were decreased. Therefore, it could be further verified that ALDBGALG0000005049-SCD induced inflammation in chicken myoblasts. Additionally, we also found the increased expressions of IL-1β, IL-6, IL-8, and CCL4 and inflammatory cell infiltration in microstructure of chicken muscles treated with Se deficiency. Thus we could conclude that ALDBGALG0000005049 regulating SCD involving in PPAR pathway played an important role in Se deficiency induced muscle injury.

In summary, we profiled the lncRNAs and mRNAs in the chicken muscle suffered from Se deficiency and the KEGG analysis revealed the PPAR pathway was the significantly dysregulated pathway. Combined with the results of lncRNA-mRNA co-expression network, we verified the regulatory relationship between ALDBGALG0000005049 and SCD. Importantly, downregulated ALDBGALG0000005049 induced inflammation response by regulating SCD might play an important role in Se deficiency induced muscle damage.

## MATERIALS AND METHODS

### Birds, diets and muscle samples collection

All procedures used in this study were approved by the Institutional Animal Care and Use Committee of Northeast Agricultural University. One hundred and eighty male broiler chicks (1 day old; Weiwei Co. Ltd., Harbin, China) were randomly divided into two groups (90 chickens per group). The chickens were maintained on either a sodium selenite diet (C group) containing 0.2 mg Se/kg or a Se-deficient diet (-Se group) containing 0.008 mg Se/kg and all the chicks were allowed ad libitum consumption of feed and water over the entire experimental period. After fed 25-30 days, the chickens were killed following euthanasia with sodium pentobarbital and pectoral muscles were quickly removed. The pectoral muscles tissues were rinsed with ice-cold sterile deionized water, frozen immediately in liquid nitrogen, and stored at −80°C until needed.

### Histological analysis of muscles

Histological analysis was performed according to previous study [6]. Briefly, the muscle tissue samples were rapidly fixed in 10% neutral buffered formalin solution for more than 24 h after chicken autopsying. Fixed samples were processed using the conventional paraffin-embedding technique. From the prepared paraffin blocks, sections were obtained and stained with hematoxylin and eosin (HE) for light microscopic examination.

### RNA isolated, library preparation and sequencing

Total RNA of each sample was isolated using Trizol reagent (Invitrogen, Carlsbad, CA, USA). Total RNA purity, concentration and integrity were checked, respectively. The library preparation and sequencing include the following steps. Firstly, ribosomal RNA was removed by Epicentre Ribo-zero™ rRNA Removal Kit (Epicentre, USA), and rRNA free residue was cleaned up by ethanol precipitation. Secondly, sequencing libraries were generated by NEBNext^®^ Ultra™ Directional RNA Library Prep Kit for Illumina^®^ (NEB, USA) using the rRNA-depleted RNA. Thirdly, double-stranded cDNA was synthesized replacing dTTPs with dUTPs in the reaction buffer used in second strand cDNA synthesis after RNA fragmenting. Fourthly, the library fragments were purified with AMPure XP system (Beckman Coulter, Beverly, USA) to select cDNA fragments of preferentially 150∼200 bp in length. Fifthly, 3 μl USER Enzyme (NEB, USA) was used with size-selected, adaptor-ligated cDNA at 37°C for 15 min followed by 5 min at 95°C. Then PCR was performed with Phusion High-Fidelity DNA polymerase, Universal PCR primers and Index (X) Primer, and products were purified (AMPure XP system) and library quality was assessed on the Agilent Bioanalyzer 2100 system. Finally, the clustering of the index-coded samples was performed on a cBot Cluster Generation System and the libraries were sequenced on an Illumina Hiseq 2000.

### Mapping to the reference genome and transcriptome assembly

After clean reads obtained, the index of the reference genome was built using Bowtie v2.0.6 and paired-end clean reads were aligned to the reference genome using TopHat v2.0.9. Then the mapped reads of each sample were assembled by both Scripture (beta2) [45] and Cufflinks (v2.1.1) [46] in a reference-based approach. In addition, Scripture was run with default parameters, Cufflinks was run with “min-frags-per-transfrag=0” and “--library-type”, other parameters were set as default.

### LncRNA and novel mRNA identification

Firstly, the reads coverage of every transcript was calculated using Cufflinks v2.1.1 [46], and those with less than 3 reads coverage were removed. Secondly, transcripts with single-exon and less than 200 bp were excluded. Thirdly, transcripts that belonged to rRNA, tRNA, snRNA, snoRNA, pre-miRNA and pseudogenes, etc. were discarded. Finally, CNCI v2 [47], CPC-0.9-r2 [48], Pfam-scan v1.3 [49], and PhyloCSF v20121028 [50] were used to evaluate the coding potential of transcripts. Transcripts that passed all the filters mentioned above were considered candidate lncRNAs, and those with coding potential were considered novel mRNAs.

### Quantification of gene expression levels and differential expression analysis

Both lncRNAs and coding genes in each sample was calculate in fragments per kilo-base of exon per million mapped fragments (FPKM) using the Cuffdiff tool in Cufflinks v2.1.1 [46]. Then Cuffdiff provides statistical routines for determining differential expression in digital transcript or gene expression data using a model based on the negative binomial distribution [46], and transcripts or genes with an P <0.05 were assigned as differentially expressed.

### Target gene prediction

Cis role is lncRNA acting on neighboring target genes. In this study, we searched coding genes 100kb upstream and downstream of lncRNA and then analyzed their function.

### GO and KEGG enrichment analysis

GO analysis of the differentially expressed mRNAs was performed using GOseq R package, in which gene length bias was corrected. GO terms with corrected P < 0.05 were considered significantly enriched by differential expressed genes. Meanwhile, KOBAS software was used to test the statistical enrichment of differential expression mRNAs in KEGG pathways.

### Cell culture and siRNA transfected

The pectorals of 12-day-old chicken embryos were minced and digested with 0.1% collagenase type I (Invitrogen, Carlsbad, CA, USA). To release single cells, the suspension was dispensed by gently pipette and filtered to remove the large debris. The cell suspension subjected to a density gradient centrifugation in three discontinuous layers with 20%, 30% and 55% Percoll (Pharmacia, Uppsala, Sweden), and the cells between the interface of 30% and 55% Percoll were harvested, and re-suspended in growth medium. Myoblasts at a density of 2×10^5^ cells/cm^2^ were seeded in six-well plates (Jet, China) covered with 0.1% gelatin (Sigma, St. Louis, MO). The six-well plates were placed in the cell incubator with growth medium in 5% at 37°C. Myoblasts proliferated for 48 h at 37°C, then the differentiation of myoblasts was induced by replacing growth medium with differentiation medium (DM).

The specific siRNA for lncRNA-ALDBGALG0000 005049 gene was designed (Genepharma, Shamghai, China) and after the chicken myoblasts were plated in six-well plates at 70-80% confluence, the cells were transfected with a final concentration of 50 nM negative control siRNA (NC group) or ALDBGALG0000005049 siRNA (siRNA group) using Lipofectamine 2000 (Invitrogen, USA). After transfection for approximately 48 h, the cells were harvested for analysis. The sequences of siRNA were listed in Table 3.

**Table 3:** List of siRNA sequences in this study

siRNA (sequence location)	Primer sequences (5′-3′)
ALDBGALG0000005049_siRNA (189)	Up: CCAAGGGUUCCUUCUGAAUTT
	Down: AUUCAGAAGGAACCCUUGGTT
Negative control	Up: UUCUCCGAACGUGUCACGUTT
	Down: ACGUGACACGUUCGGAGAATT

### RNA isolation and qPCR

The total RNA was extracted from chicken muscles tissues and chicken myoblasts using Trizol reagent according to the manufacturer's instructions (Invitrogen, Shanghai, China). Complementary DNA (cDNA) was made Revert Aid first strand cDNA synthesis kit (Thermo Scientific, MA, USA), following the manufacturer's protocols. The β-actin as housekeeping gene was used as an internal reference, and chicken lncRNAs and mRNAs primer sequences were detailed in Supplementary Table 3. The relative expression levels of lncRNAs and mRNAs were evaluated by the 2^−ΔΔCt^ method.

### Protein extraction and western blotting

Chicken muscles and chicken myoblasts protein extracts were subjected to SDS-polyacrylamide gel electrophoresis under reducing conditions on 15% gels. Separated proteins were then transferred to nitrocellulose membranes using tank transfer for 1 h at 100 mA in Tris–glycine buffer containing 20% methanol. The membranes were blocked with 5% skim milk for 18 h and incubated overnight with diluted primary antibody against IL-1β, IL-6, IL-8, and CCL4 (1 : 1000, Abcam, China), followed by a horseradish peroxidase (HRP)-conjugated secondary antibody against rabbit IgG (1 : 1000, Santa Cruz Biotechnology, USA). To verify equal sample loading, the membrane was incubated with a monoclonal β-actin antibody (1 : 1000, Santa Cruz Biotechnology, USA), followed by an HRP-conjugated goat anti-mouse IgG (1 : 1000). The signal was detected using an enhanced chemiluminescence system (Cheml Scope5300, Clinx Science Instruments, Shanghai, China). The optical density (OD) of each band was determined using the Image VCD gel imaging system.

### Statistical analysis

The data shown below are the mean ± SD of three independent experiments. The differences were considered statistically significant at p<0.05, unpaired or paired two-tailed t-test was used for individual comparisons and one-way analysis of variance (ANOVA) followed by Duncan's test was used for multiple comparisons. All the analyses were performed with the SPSS software (version 19.0) (IBM Corporation, New York, NY, USA).

## SUPPLEMENTARY TABLES







## Abbreviations

LncRNAs: Long non-coding RNAs
Se: Selenium
PPAR: Peroxisome Proliferator-Activated Receptor
SCD: stearoyl-CoA desaturase
IL: interleukin
CCL4: chemokine (C-C motif) ligand 4
DMD: Duchenne Muscular Dystrophy
SFA: saturated fatty acids
GO: Gene ontology
KEGG: Kyoto Encyclopedia of Genes and Genomes
qPCR: Quantitative PCR
PDXK: pyridoxal (pyridoxine, vitamin B6) kinase
S100A9: S100 calcium binding protein A9
LRRC4C: leucine rich repeat containing 4C
DCX: doublecortin
SLC51A: solute carrier family 51 alpha subunit
MTTP: microsomal triglyceride transfer protein
siRNA: small interfering RNA
HE: hematoxylin and eosin
FPKM: exon per million mapped fragments
DM: differentiation medium
cDNA: Complementary DNA
OD: optical density
ANOVA: one-way analysis of variance
