# Supplementary material for: Downregulated long noncoding RNA ALDBGALG0000005049 induces inflammation in chicken muscle suffered from selenium deficiency by regulating stearoyl-CoA desaturase
Source: Oncotarget. 2017 Apr 18;8(32):52761–74. doi: 10.18632/oncotarget.17187 (PMC5581067; doi:10.18632/oncotarget.17187)
Supplement: Supplementary file 1 [file oncotarget-08-52761-s001.pdf]

Downregulated long noncoding RNA ALDBGALG0000005049 induces inflammation in chicken muscle suffered from selenium deficiency by regulating stearoyl-CoA desaturase

SUPPLEMENTARY TABLES

Supplementary Table 1: The differently expression of lncRNAs and mRNAs

See Supplementary File 1

Supplementary Table 2: The potential targets of lncRNAs were predicted in cis

See Supplementary File 2

Supplementary Table 3: The primers used in the present study

Gene	Forward primer	Reverse primer
ALDBGALG0000000325	5'-GCTGTGATTTCCTCCTCTGG-3'	5'-TCACAGTGCCCTCATCACTC-3'
ALDBGALG0000003187	5'-CAGTGCTCATCGCTTACCAG-3'	5'-GCACAGGTGGAAAGAACTCA-3'
ALDBGALG0000004022	5'-GCCTGTTAAGGGATGATGGA-3'	5'-GAGCAGGGACTCACAGGAAG-3'
ALDBGALG0000005049	5'-TGACACCAAGGGTTCCTTCT-3'	5'-AAAGTGACTGGGCAAACACC-3'
ALDBGALG0000005593	5'-CTTGCTTCTGTCCTGGGTGT-3'	5'-ATCTGCAGTGTTCAGCATCG-3'
XLOC_1170550	5'-TTGTCACCCAGTGTGTTCAGA-3'	5'-ACGGCACAAAGGACTGAAAG-3'
XLOC_1196171	5'-CTCTCTGCCACTGCTGTACG-3'	5'-CTACCGTTCTCTCCGTTTGC-3'
PDXK	5'-ATCTACAGGCACCGCTAGGA-3'	5'-GGCAGCATCCACTTTAGGAG-3'
S100A9	5'-TTGGTGAAGTGATGCTCCTG-3'	5'-AGTGGTTGTGCTGATGTTGG-3'
DCX	5'-GCACACGTATGAATGGGTTG-3'	5'-GTTGCGATAGAAACGCACCT-3'
SCD	5'-TACGCAAACACCCAGATGTC-3'	5'-AGCACAGCAACACCACTGAG-3'
SLC51A	5'-AGATTCTCCTCCTGCTGACG-3'	5'-GCTGCATGTTCATCTGCTGT-3'
MTTP	5'-CATCCCAGTGAAATGCTCCT-3'	5'-TCACACAGCTTTCGGATGAG-3'
LRRC4C	5'-AGAGTTCGGATTGCTGTGCT-3'	5'-TGGCAGAAGCTGTGGTATTG-3'
PPARα	5'-ATCCCTGGCTTCTCCAATCT-3'	5'-TAGGCTACCAGCATCCCATC-3'
PPARδ	5'-CATGGAGCCCAAGTTTGAGT-3'	5'-GGACGATCTCCACACAGGAT-3'
PPARγ	5'-CACAAGCGGAGAAGGAGAAG-3'	5'-TCAGCGGGAAGGACTTTATG-3'
β-actin	5'-CCGCTCTATGAA GGCTACGC -3'	5'-CTCTCG GCTGTGGTGGTGAA-3'
