# Supplementary material for: Downregulated long noncoding RNA ALDBGALG0000005049 induces inflammation in chicken muscle suffered from selenium deficiency by regulating stearoyl-CoA desaturase
Source: Oncotarget. 2017 Apr 18;8(32):52761–74. doi: 10.18632/oncotarget.17187 (PMC5581067; doi:10.18632/oncotarget.17187)
Supplement: Supplementary file 2 [file oncotarget-08-52761-s002.doc]

**Supplementary Table 1: The differently expression of lncRNAs and mRNAs**

| Transcript_id | Gene_id | Length | -SeM_FPKM | CM_FPKM | Log2(foldchange) | Pvalue |
| --- | --- | --- | --- | --- | --- | --- |
| XM_004950023.1 | 100857891.0000 | 476.0000 | 0.2051 | 39.6813 | -7.5960 | 0.0055 |
| NM_204688.2 | 395423.0000 | 1193.0000 | 0.2709 | 24.3113 | -6.4875 | 0.0001 |
| NM_001277417.1 | 424822.0000 | 651.0000 | 0.2526 | 18.4524 | -6.1911 | 0.0027 |
| NM_001197038.1 | 419498.0000 | 1340.0000 | 0.4695 | 31.9948 | -6.0906 | 0.0001 |
| XM_425505.3 | 427933.0000 | 1898.0000 | 0.0997 | 4.7687 | -5.5801 | 0.0001 |
| NM_204192.3 | 374015.0000 | 526.0000 | 1.7355 | 78.4068 | -5.4976 | 0.0014 |
| XM_422811.4 | 425007.0000 | 5776.0000 | 0.0783 | 3.2396 | -5.3706 | 0.0001 |
| NM_001267719.1 | 100859435.0000 | 870.0000 | 0.0876 | 3.3853 | -5.2720 | 0.0116 |
| NM_204365.1 | 378789.0000 | 2892.0000 | 0.0637 | 2.2062 | -5.1137 | 0.0001 |
| XM_003643590.2 | 100857939.0000 | 2383.0000 | 0.3686 | 12.6988 | -5.1064 | 0.0001 |
| XM_421341.4 | 423432.0000 | 1497.0000 | 0.0759 | 2.5670 | -5.0807 | 0.0030 |
| NM_001103202.1 | 425200.0000 | 912.0000 | 1.0704 | 36.1739 | -5.0787 | 0.0001 |
| XM_003641761.2 | 100859876.0000 | 1858.0000 | 0.2320 | 7.6079 | -5.0354 | 0.0001 |
| NM_001145489.1 | 416980.0000 | 447.0000 | 1.2696 | 40.2370 | -4.9861 | 0.0001 |
| XM_422715.4 | 424904.0000 | 11242.0000 | 0.1696 | 5.3143 | -4.9700 | 0.0001 |
| NM_001277721.1 | 422060.0000 | 2345.0000 | 0.1134 | 3.3097 | -4.8673 | 0.0001 |
| NM_001277625.1 | 427531.0000 | 896.0000 | 0.4016 | 10.9141 | -4.7642 | 0.0001 |
| XM_426667.4 | 429111.0000 | 2046.0000 | 0.0943 | 2.4213 | -4.6817 | 0.0001 |
| NM_205335.2 | 396277.0000 | 657.0000 | 1.4887 | 36.4315 | -4.6130 | 0.0001 |
| NM_204938.1 | 395780.0000 | 1197.0000 | 1.6838 | 37.3899 | -4.4729 | 0.0001 |
| Novel000364.1 | Novel000364 | 417.0000 | 0.2687 | 5.9600 | -4.4714 | 0.0084 |
| NM_205513.1 | 396519.0000 | 2806.0000 | 0.2102 | 4.6450 | -4.4656 | 0.0001 |
| NM_001257214.1 | 416682.0000 | 1381.0000 | 0.3126 | 6.6679 | -4.4149 | 0.0001 |
| XM_422360.4 | 424523.0000 | 2854.0000 | 0.1584 | 3.3247 | -4.3917 | 0.0001 |
| NM_204541.2 | 395220.0000 | 789.0000 | 1.2026 | 25.1374 | -4.3856 | 0.0001 |
| XM_004948718.1 | 101749387.0000 | 459.0000 | 0.2188 | 4.5608 | -4.3818 | 0.0055 |
| NM_001277981.1 | 420045.0000 | 1715.0000 | 0.1623 | 3.3689 | -4.3753 | 0.0001 |
| NM_001278004.1 | 421740.0000 | 1399.0000 | 0.1101 | 2.2740 | -4.3678 | 0.0003 |
| NM_001032390.2 | 419486.0000 | 978.0000 | 2.8505 | 58.7683 | -4.3658 | 0.0002 |
| XM_003640446.2 | 100857124.0000 | 935.0000 | 0.3100 | 6.3781 | -4.3627 | 0.0045 |
| XM_001233805.3 | 770471.0000 | 1825.0000 | 0.3169 | 6.4893 | -4.3561 | 0.0001 |
| NM_204989.1 | 395837.0000 | 1531.0000 | 1.3911 | 27.9856 | -4.3304 | 0.0001 |
| NM_205356.2 | 396307.0000 | 3075.0000 | 0.9781 | 19.1055 | -4.2878 | 0.0001 |
| XM_419273.3 | 421195.0000 | 7872.0000 | 0.0628 | 1.2051 | -4.2625 | 0.0001 |
| NM_205385.1 | 396345.0000 | 860.0000 | 0.2449 | 4.6043 | -4.2328 | 0.0004 |
| Novel002897.1 | Novel002897 | 3633.0000 | 0.0778 | 1.4153 | -4.1848 | 0.0001 |
| NM_001277382.1 | 423916.0000 | 1640.0000 | 0.9567 | 17.3407 | -4.1799 | 0.0001 |
| NM_205384.1 | 396344.0000 | 637.0000 | 6.1660 | 111.5980 | -4.1778 | 0.0001 |
| XM_424435.4 | 426826.0000 | 2509.0000 | 0.1500 | 2.6475 | -4.1418 | 0.0001 |
| XM_001233227.3 | 771012.0000 | 1771.0000 | 0.2626 | 4.6148 | -4.1351 | 0.0001 |
| NM_001252256.1 | 416683.0000 | 1334.0000 | 0.6531 | 11.2871 | -4.1111 | 0.0001 |
| NM_001044644.1 | 419848.0000 | 2585.0000 | 0.2435 | 4.2021 | -4.1093 | 0.0001 |
| NM_001001751.2 | 414832.0000 | 1637.0000 | 0.3213 | 5.3197 | -4.0494 | 0.0001 |
| NM_204882.1 | 395696.0000 | 1455.0000 | 0.3594 | 5.7012 | -3.9876 | 0.0001 |
| XM_416822.3 | 418623.0000 | 2965.0000 | 0.1634 | 2.5679 | -3.9737 | 0.0001 |
| NM_001001473.2 | 414140.0000 | 1681.0000 | 5.5375 | 86.5170 | -3.9657 | 0.0001 |
| NM_001277492.1 | 423433.0000 | 1711.0000 | 0.1817 | 2.8373 | -3.9651 | 0.0001 |
| NM_001001616.1 | 414746.0000 | 2839.0000 | 0.3427 | 5.3470 | -3.9637 | 0.0001 |
| XM_003642539.2 | 100857116.0000 | 958.0000 | 0.9947 | 15.2789 | -3.9411 | 0.0001 |
| XM_416418.4 | 418187.0000 | 3367.0000 | 0.0851 | 1.2976 | -3.9299 | 0.0001 |
| NM_207180.1 | 395364.0000 | 1655.0000 | 2.5782 | 38.9438 | -3.9169 | 0.0001 |
| XM_004949786.1 | 100859777.0000 | 1968.0000 | 0.0659 | 0.9904 | -3.9102 | 0.0029 |
| NM_001012564.1 | 421292.0000 | 1506.0000 | 0.3516 | 5.2723 | -3.9066 | 0.0001 |
| XM_422502.3 | 424669.0000 | 3936.0000 | 0.1492 | 2.1617 | -3.8572 | 0.0001 |
| XM_416207.4 | 417968.0000 | 3871.0000 | 0.0682 | 0.9653 | -3.8226 | 0.0001 |
| XM_003640448.2 | 769009.0000 | 747.0000 | 0.2843 | 4.0103 | -3.8184 | 0.0001 |
| XM_417046.4 | 418851.0000 | 1374.0000 | 0.0822 | 1.1294 | -3.7800 | 0.0002 |
| NM_205261.1 | 396197.0000 | 1892.0000 | 11.2987 | 149.5530 | -3.7264 | 0.0001 |
| XM_003643132.2 | 100859713.0000 | 1322.0000 | 0.1040 | 1.3737 | -3.7236 | 0.0036 |
| XM_003641502.2 | 423943.0000 | 12931.0000 | 0.1119 | 1.4693 | -3.7149 | 0.0001 |
| NM_204584.1 | 395276.0000 | 1339.0000 | 1.5419 | 19.8073 | -3.6833 | 0.0001 |
| XM_421513.4 | 423629.0000 | 1215.0000 | 0.2908 | 3.6266 | -3.6406 | 0.0001 |
| NM_001277917.1 | 430670.0000 | 881.0000 | 1.5000 | 18.4779 | -3.6228 | 0.0001 |
| XM_001234140.3 | 770818.0000 | 465.0000 | 1.1152 | 13.7193 | -3.6209 | 0.0001 |
| NM_001159315.1 | 396459.0000 | 333.0000 | 29.9342 | 363.1760 | -3.6008 | 0.0001 |
| XM_419618.4 | 421580.0000 | 2930.0000 | 0.4128 | 5.0042 | -3.5996 | 0.0001 |
| NM_001109784.1 | 422709.0000 | 2698.0000 | 0.2980 | 3.6007 | -3.5949 | 0.0018 |
| NM_205061.1 | 395935.0000 | 1575.0000 | 0.8140 | 9.7267 | -3.5789 | 0.0001 |
| NM_001277493.1 | 423434.0000 | 1438.0000 | 0.3453 | 3.9843 | -3.5283 | 0.0001 |
| NM_204433.1 | 395076.0000 | 1902.0000 | 0.1572 | 1.8127 | -3.5275 | 0.0001 |
| XM_416421.4 | 418190.0000 | 2631.0000 | 0.1424 | 1.5997 | -3.4894 | 0.0001 |
| NM_001144849.2 | 431585.0000 | 2973.0000 | 0.1460 | 1.5570 | -3.4147 | 0.0001 |
| XM_426327.3 | 428771.0000 | 3849.0000 | 0.2091 | 2.1947 | -3.3917 | 0.0001 |
| NM_001167683.1 | 373926.0000 | 1839.0000 | 2.3861 | 25.0034 | -3.3894 | 0.0001 |
| NM_001277994.1 | 417431.0000 | 1385.0000 | 0.7845 | 8.1053 | -3.3690 | 0.0001 |
| XM_420925.4 | 422993.0000 | 518.0000 | 0.1820 | 1.8611 | -3.3540 | 0.0094 |
| NM_001130741.1 | 419110.0000 | 2912.0000 | 0.1840 | 1.8412 | -3.3229 | 0.0001 |
| NM_001199501.1 | 420342.0000 | 1217.0000 | 0.0972 | 0.9638 | -3.3105 | 0.0006 |
| NM_001040463.1 | 418826.0000 | 1470.0000 | 0.1022 | 1.0026 | -3.2938 | 0.0016 |
| NM_001277636.1 | 431235.0000 | 847.0000 | 2.7370 | 25.4471 | -3.2168 | 0.0001 |
| XM_416629.2 | 418414.0000 | 1067.0000 | 0.1093 | 1.0158 | -3.2160 | 0.0055 |
| XM_004944907.1 | 416235.0000 | 1703.0000 | 3.2621 | 29.8639 | -3.1945 | 0.0001 |
| NM_001012997.1 | 417165.0000 | 1762.0000 | 0.5499 | 4.9958 | -3.1834 | 0.0001 |
| NM_001001611.2 | 414343.0000 | 685.0000 | 0.7290 | 6.4191 | -3.1383 | 0.0001 |
| XM_001234022.3 | 771806.0000 | 1221.0000 | 1.1146 | 9.6646 | -3.1162 | 0.0001 |
| XM_003643131.2 | 426827.0000 | 1385.0000 | 0.0984 | 0.8476 | -3.1062 | 0.0073 |
| NM_205442.1 | 396423.0000 | 2578.0000 | 0.2177 | 1.8565 | -3.0924 | 0.0001 |
| NM_205238.1 | 396166.0000 | 796.0000 | 0.7848 | 6.6166 | -3.0756 | 0.0001 |
| XM_419601.3 | 421560.0000 | 1211.0000 | 0.2649 | 2.2143 | -3.0636 | 0.0001 |
| XM_004942118.1 | 101750594.0000 | 718.0000 | 0.2221 | 1.8050 | -3.0224 | 0.0094 |
| XM_004948077.1 | 100859721.0000 | 415.0000 | 1.8256 | 14.6204 | -3.0015 | 0.0001 |
| XM_415247.4 | 395496.0000 | 4047.0000 | 0.4632 | 3.6866 | -2.9926 | 0.0001 |
| Novel000643.1 | Novel000643 | 1497.0000 | 0.4107 | 3.1650 | -2.9459 | 0.0001 |
| XM_420819.4 | 422876.0000 | 3198.0000 | 0.0755 | 0.5812 | -2.9441 | 0.0020 |
| NM_001145491.1 | 771102.0000 | 348.0000 | 0.7028 | 5.3097 | -2.9174 | 0.0080 |
| NM_205019.2 | 395873.0000 | 2007.0000 | 1.0001 | 7.4996 | -2.9066 | 0.0001 |
| XM_419749.4 | 421712.0000 | 1638.0000 | 0.1494 | 1.0660 | -2.8346 | 0.0002 |
| NM_001277698.1 | 770795.0000 | 1180.0000 | 0.7979 | 5.6059 | -2.8126 | 0.0001 |
| NM_204349.2 | 374267.0000 | 1550.0000 | 0.3480 | 2.3494 | -2.7550 | 0.0001 |
| XM_001234581.3 | 423101.0000 | 10098.0000 | 0.6547 | 4.4130 | -2.7528 | 0.0001 |
| NM_205168.3 | 396080.0000 | 836.0000 | 0.4313 | 2.8728 | -2.7358 | 0.0001 |
| XM_419410.4 | 421349.0000 | 5636.0000 | 0.1817 | 1.1952 | -2.7177 | 0.0001 |
| XM_004950351.1 | 100858140.0000 | 339.0000 | 1.9033 | 11.8054 | -2.6328 | 0.0005 |
| NM_001044633.1 | 396535.0000 | 14150.0000 | 1.7802 | 10.7707 | -2.5970 | 0.0001 |
| NM_204343.1 | 374258.0000 | 1416.0000 | 0.2034 | 1.2199 | -2.5840 | 0.0005 |
| XM_004941926.1 | 772339.0000 | 2661.0000 | 0.2619 | 1.5363 | -2.5524 | 0.0002 |
| XM_416940.3 | 418741.0000 | 2224.0000 | 1.7518 | 10.2222 | -2.5448 | 0.0005 |
| NM_001277590.1 | 424607.0000 | 1353.0000 | 0.3485 | 2.0057 | -2.5249 | 0.0001 |
| NM_001199495.1 | 420225.0000 | 2976.0000 | 0.3240 | 1.8631 | -2.5237 | 0.0001 |
| XM_004949783.1 | 101748790.0000 | 2308.0000 | 0.1154 | 0.6490 | -2.4918 | 0.0005 |
| XM_419257.4 | 421177.0000 | 2043.0000 | 0.2384 | 1.2989 | -2.4459 | 0.0001 |
| NM_001006278.1 | 418983.0000 | 878.0000 | 0.2512 | 1.3538 | -2.4301 | 0.0018 |
| XM_422368.4 | 424531.0000 | 2313.0000 | 0.1602 | 0.8324 | -2.3772 | 0.0001 |
| XM_003641585.2 | 100859223.0000 | 3054.0000 | 0.5117 | 2.6466 | -2.3708 | 0.0001 |
| XM_425404.3 | 427830.0000 | 2850.0000 | 0.1205 | 0.6182 | -2.3591 | 0.0002 |
| XM_001233369.3 | 770055.0000 | 4122.0000 | 0.2717 | 1.3925 | -2.3575 | 0.0001 |
| XM_417267.4 | 419076.0000 | 1290.0000 | 6.0401 | 30.6321 | -2.3424 | 0.0001 |
| NM_001001298.1 | 408024.0000 | 1544.0000 | 0.1267 | 0.6339 | -2.3224 | 0.0015 |
| Novel002578.1 | Novel002578 | 5370.0000 | 0.1290 | 0.6412 | -2.3128 | 0.0001 |
| XM_003641458.2 | 423680.0000 | 4131.0000 | 0.1515 | 0.7315 | -2.2719 | 0.0001 |
| XM_422225.4 | 424380.0000 | 1700.0000 | 0.1826 | 0.8775 | -2.2649 | 0.0027 |
| NM_204182.1 | 374002.0000 | 1233.0000 | 0.4200 | 1.9964 | -2.2491 | 0.0001 |
| XM_004950214.1 | 101749313.0000 | 448.0000 | 0.3959 | 1.8204 | -2.2012 | 0.0041 |
| NM_001252254.1 | 416012.0000 | 2580.0000 | 0.2333 | 1.0684 | -2.1949 | 0.0002 |
| XM_004938532.1 | 418741.0000 | 6824.0000 | 0.6025 | 2.7438 | -2.1871 | 0.0110 |
| XM_414669.3 | 416353.0000 | 5890.0000 | 0.2285 | 1.0224 | -2.1619 | 0.0001 |
| XM_426083.4 | 428529.0000 | 5218.0000 | 0.1862 | 0.8145 | -2.1289 | 0.0001 |
| XM_004935373.1 | 771916.0000 | 3386.0000 | 0.2192 | 0.9534 | -2.1208 | 0.0012 |
| XM_421579.4 | 423698.0000 | 4729.0000 | 0.2471 | 1.0717 | -2.1168 | 0.0001 |
| XM_422282.4 | 424440.0000 | 1818.0000 | 0.7063 | 3.0486 | -2.1097 | 0.0001 |
| XM_003640356.2 | 100858458.0000 | 2464.0000 | 0.1291 | 0.5568 | -2.1083 | 0.0005 |
| NM_001012555.2 | 420280.0000 | 2411.0000 | 0.2851 | 1.2218 | -2.0996 | 0.0001 |
| NM_001039258.2 | 415860.0000 | 3595.0000 | 0.4101 | 1.7365 | -2.0823 | 0.0001 |
| XM_004950138.1 | 101749159.0000 | 399.0000 | 0.8211 | 3.4768 | -2.0821 | 0.0134 |
| XM_417988.4 | 419861.0000 | 1648.0000 | 0.3430 | 1.4401 | -2.0698 | 0.0001 |
| NM_001030576.2 | 415820.0000 | 1880.0000 | 0.2359 | 0.9646 | -2.0316 | 0.0002 |
| NM_205347.1 | 396291.0000 | 2757.0000 | 0.3348 | 1.3622 | -2.0248 | 0.0001 |
| XM_004949173.1 | 101751815.0000 | 3765.0000 | 0.1612 | 0.6471 | -2.0051 | 0.0002 |
| NM_204683.1 | 395418.0000 | 2910.0000 | 0.3104 | 1.2295 | -1.9857 | 0.0001 |
| XM_001232057.3 | 769339.0000 | 2524.0000 | 0.7406 | 2.9196 | -1.9790 | 0.0051 |
| NM_001011690.3 | 422633.0000 | 615.0000 | 0.7032 | 2.7581 | -1.9717 | 0.0025 |
| XM_421662.4 | 423790.0000 | 2037.0000 | 20.5907 | 80.3555 | -1.9644 | 0.0001 |
| NM_207178.1 | 396272.0000 | 2638.0000 | 0.4002 | 1.5611 | -1.9639 | 0.0001 |
| XM_004938740.1 | 418892.0000 | 5840.0000 | 2.6350 | 9.7798 | -1.8920 | 0.0001 |
| NM_001001470.1 | 395708.0000 | 1344.0000 | 1.2383 | 4.5882 | -1.8896 | 0.0001 |
| NM_204605.1 | 395306.0000 | 2988.0000 | 1.0045 | 3.6840 | -1.8748 | 0.0001 |
| XM_004938217.1 | 418359.0000 | 6450.0000 | 0.1939 | 0.7070 | -1.8666 | 0.0117 |
| XM_003641514.2 | 100858647.0000 | 590.0000 | 6.5731 | 23.3444 | -1.8284 | 0.0001 |
| XM_421698.4 | 423828.0000 | 5662.0000 | 0.1885 | 0.6541 | -1.7953 | 0.0001 |
| XM_425893.4 | 428333.0000 | 1834.0000 | 0.2184 | 0.7498 | -1.7794 | 0.0037 |
| XM_420607.4 | 422653.0000 | 3486.0000 | 0.2022 | 0.6899 | -1.7705 | 0.0071 |
| NM_001278073.1 | 418411.0000 | 523.0000 | 0.4859 | 1.6514 | -1.7650 | 0.0128 |
| XM_415405.4 | 417122.0000 | 5133.0000 | 0.4147 | 1.3966 | -1.7517 | 0.0001 |
| NM_204242.1 | 374089.0000 | 2601.0000 | 0.2308 | 0.7750 | -1.7475 | 0.0004 |
| XM_004940918.1 | 422427.0000 | 7556.0000 | 0.8089 | 2.7092 | -1.7438 | 0.0003 |
| NM_204609.1 | 395313.0000 | 2487.0000 | 7.6127 | 25.2983 | -1.7326 | 0.0001 |
| XM_001235179.3 | 771974.0000 | 1881.0000 | 0.6947 | 2.2525 | -1.6970 | 0.0001 |
| XM_423439.4 | 425711.0000 | 628.0000 | 1.0298 | 3.3376 | -1.6965 | 0.0014 |
| NM_001077233.1 | 426611.0000 | 5111.0000 | 0.7975 | 2.5539 | -1.6791 | 0.0001 |
| NM_205060.1 | 395933.0000 | 2262.0000 | 0.2081 | 0.6525 | -1.6489 | 0.0035 |
| XM_001236844.3 | 777386.0000 | 1167.0000 | 0.3368 | 1.0434 | -1.6311 | 0.0017 |
| XM_417501.3 | 419333.0000 | 3852.0000 | 0.4354 | 1.3359 | -1.6173 | 0.0012 |
| XM_001235520.3 | 772374.0000 | 1652.0000 | 0.3263 | 0.9980 | -1.6129 | 0.0005 |
| XM_416398.3 | 418167.0000 | 2615.0000 | 3.6198 | 10.9484 | -1.5967 | 0.0019 |
| NM_205304.1 | 396241.0000 | 2377.0000 | 15.3018 | 45.9741 | -1.5871 | 0.0001 |
| NM_204113.2 | 373902.0000 | 1835.0000 | 0.1991 | 0.5931 | -1.5745 | 0.0048 |
| NM_001031188.1 | 423164.0000 | 3125.0000 | 0.2055 | 0.6060 | -1.5604 | 0.0005 |
| NM_001033642.1 | 419851.0000 | 2753.0000 | 3.4295 | 9.9841 | -1.5416 | 0.0001 |
| XM_004945147.1 | 416479.0000 | 5608.0000 | 0.2211 | 0.6367 | -1.5261 | 0.0040 |
| NM_204890.1 | 395706.0000 | 1420.0000 | 2.9190 | 8.3417 | -1.5149 | 0.0001 |
| XM_004935941.1 | 101751539.0000 | 1193.0000 | 0.2091 | 0.5975 | -1.5146 | 0.0121 |
| NM_001001296.4 | 403120.0000 | 478.0000 | 53.2924 | 151.9540 | -1.5116 | 0.0001 |
| XM_417990.4 | 419863.0000 | 1574.0000 | 0.4006 | 1.1370 | -1.5049 | 0.0013 |
| XM_426208.3 | 428650.0000 | 1418.0000 | 2.4779 | 6.9874 | -1.4957 | 0.0001 |
| XM_418831.4 | 420732.0000 | 2847.0000 | 0.5553 | 1.5578 | -1.4881 | 0.0001 |
| XM_004938051.1 | 419096.0000 | 2482.0000 | 0.4902 | 1.3391 | -1.4497 | 0.0010 |
| XM_419937.4 | 421921.0000 | 3310.0000 | 4.5256 | 12.2987 | -1.4423 | 0.0001 |
| XM_004941122.1 | 100857411.0000 | 568.0000 | 4.2114 | 11.3039 | -1.4245 | 0.0016 |
| XM_004949834.1 | 101750175.0000 | 1273.0000 | 0.2462 | 0.6605 | -1.4235 | 0.0111 |
| XM_004942508.1 | 101749922.0000 | 955.0000 | 0.3867 | 1.0324 | -1.4169 | 0.0114 |
| XM_004942880.1 | 426214.0000 | 1548.0000 | 0.4467 | 1.1927 | -1.4169 | 0.0049 |
| XM_004936634.1 | 424517.0000 | 2576.0000 | 1.9874 | 5.2986 | -1.4148 | 0.0105 |
| NM_001007977.2 | 427308.0000 | 1590.0000 | 9.2695 | 24.6955 | -1.4137 | 0.0001 |
| XM_004941189.1 | 428761.0000 | 2287.0000 | 0.2803 | 0.7439 | -1.4080 | 0.0011 |
| NM_001277697.1 | 424916.0000 | 1285.0000 | 0.5305 | 1.4046 | -1.4049 | 0.0054 |
| XM_004939297.1 | 420559.0000 | 6308.0000 | 2.6686 | 6.8559 | -1.3612 | 0.0001 |
| XM_418228.4 | 420107.0000 | 2135.0000 | 8.5400 | 21.6851 | -1.3444 | 0.0001 |
| XM_004943469.1 | 424983.0000 | 4155.0000 | 0.5648 | 1.4263 | -1.3366 | 0.0100 |
| NM_001199476.1 | 420198.0000 | 795.0000 | 0.8740 | 2.1920 | -1.3266 | 0.0032 |
| XM_001233004.3 | 769716.0000 | 2040.0000 | 1.2616 | 3.1560 | -1.3229 | 0.0001 |
| NM_205041.1 | 395908.0000 | 1514.0000 | 8.5423 | 21.2747 | -1.3164 | 0.0001 |
| XM_420008.4 | 422000.0000 | 2362.0000 | 1.2317 | 3.0424 | -1.3046 | 0.0001 |
| NM_213577.1 | 404755.0000 | 793.0000 | 5.3597 | 13.1595 | -1.2959 | 0.0001 |
| XM_004939102.1 | 425984.0000 | 8422.0000 | 0.5940 | 1.4573 | -1.2948 | 0.0025 |
| XM_415868.3 | 417624.0000 | 2803.0000 | 0.4726 | 1.1573 | -1.2922 | 0.0022 |
| NM_204775.1 | 395550.0000 | 1052.0000 | 68.7446 | 166.3230 | -1.2747 | 0.0001 |
| NM_204964.1 | 395811.0000 | 1750.0000 | 0.3798 | 0.9081 | -1.2578 | 0.0037 |
| NM_001171768.1 | 421019.0000 | 2310.0000 | 0.7337 | 1.7273 | -1.2352 | 0.0007 |
| NM_205046.1 | 395914.0000 | 860.0000 | 1.3372 | 3.1462 | -1.2344 | 0.0049 |
| XM_003643736.2 | 427737.0000 | 1750.0000 | 0.5482 | 1.2854 | -1.2296 | 0.0020 |
| XM_001234229.3 | 770916.0000 | 600.0000 | 1.4895 | 3.4799 | -1.2243 | 0.0046 |
| NM_204441.1 | 395085.0000 | 1302.0000 | 0.4472 | 1.0411 | -1.2191 | 0.0097 |
| XM_003642294.2 | 100859070.0000 | 4590.0000 | 0.2916 | 0.6778 | -1.2170 | 0.0013 |
| XM_003643684.2 | 100857553.0000 | 2156.0000 | 0.2409 | 0.5562 | -1.2070 | 0.0108 |
| NM_001277678.1 | 100859733.0000 | 881.0000 | 0.9671 | 2.2187 | -1.1979 | 0.0050 |
| XM_004941440.1 | 395087.0000 | 2803.0000 | 0.8194 | 1.8742 | -1.1937 | 0.0006 |
| XM_004950190.1 | 101750793.0000 | 1312.0000 | 3.1583 | 7.2221 | -1.1933 | 0.0001 |
| XM_004935841.1 | 421998.0000 | 3972.0000 | 0.4803 | 1.0976 | -1.1924 | 0.0115 |
| XM_004937774.1 | 771315.0000 | 4859.0000 | 1.6567 | 3.7737 | -1.1877 | 0.0154 |
| XM_416863.3 | 418667.0000 | 3590.0000 | 1.5033 | 3.4226 | -1.1869 | 0.0001 |
| XM_004934403.1 | 416984.0000 | 9447.0000 | 0.5630 | 1.2795 | -1.1844 | 0.0078 |
| XM_004938136.1 | 101747965.0000 | 2242.0000 | 0.4306 | 0.9757 | -1.1800 | 0.0025 |
| NM_204176.1 | 373995.0000 | 2417.0000 | 0.5049 | 1.1418 | -1.1773 | 0.0024 |
| XM_421399.4 | 423496.0000 | 2091.0000 | 0.3057 | 0.6868 | -1.1678 | 0.0084 |
| XM_004942637.1 | 395571.0000 | 3986.0000 | 0.2398 | 0.5383 | -1.1665 | 0.0035 |
| XM_425539.4 | 427967.0000 | 6632.0000 | 0.3290 | 0.7354 | -1.1604 | 0.0005 |
| XM_004950455.1 | 101750307.0000 | 1702.0000 | 0.4801 | 1.0727 | -1.1598 | 0.0058 |
| NM_001038586.1 | 428642.0000 | 2502.0000 | 0.3113 | 0.6956 | -1.1597 | 0.0041 |
| NM_001004403.2 | 422902.0000 | 1807.0000 | 1.7012 | 3.7875 | -1.1547 | 0.0002 |
| NM_001245982.1 | 428149.0000 | 1409.0000 | 1.2851 | 2.8466 | -1.1473 | 0.0011 |
| XM_419552.4 | 421507.0000 | 3080.0000 | 2.5378 | 5.6166 | -1.1461 | 0.0001 |
| XM_004935847.1 | 422002.0000 | 3027.0000 | 0.6248 | 1.3682 | -1.1309 | 0.0090 |
| XM_003641374.2 | 100857954.0000 | 2942.0000 | 7.6509 | 16.7127 | -1.1272 | 0.0001 |
| NM_001030777.1 | 418294.0000 | 2650.0000 | 25.2360 | 55.0883 | -1.1263 | 0.0001 |
| XM_420149.3 | 422148.0000 | 2671.0000 | 0.7491 | 1.6262 | -1.1183 | 0.0021 |
| XM_416158.3 | 417917.0000 | 1466.0000 | 0.3620 | 0.7847 | -1.1162 | 0.0154 |
| NM_205405.1 | 396370.0000 | 4058.0000 | 7.0665 | 15.2675 | -1.1114 | 0.0001 |
| XM_001231916.3 | 769726.0000 | 1165.0000 | 1.0875 | 2.3473 | -1.1100 | 0.0051 |
| XM_001235233.3 | 772032.0000 | 3189.0000 | 0.6176 | 1.3240 | -1.1002 | 0.0057 |
| XM_420476.4 | 422513.0000 | 4206.0000 | 5.8159 | 12.4613 | -1.0994 | 0.0059 |
| XM_003641983.2 | 100858703.0000 | 1711.0000 | 0.3771 | 0.8056 | -1.0952 | 0.0129 |
| XM_422110.4 | 424263.0000 | 3111.0000 | 2.8917 | 6.0758 | -1.0712 | 0.0003 |
| XM_417033.3 | 418837.0000 | 1651.0000 | 3.7796 | 7.8670 | -1.0576 | 0.0005 |
| XM_004943699.1 | 100857563.0000 | 2808.0000 | 0.8302 | 1.7216 | -1.0522 | 0.0059 |
| NM_205446.1 | 396430.0000 | 1441.0000 | 146.8030 | 302.9790 | -1.0453 | 0.0004 |
| XM_424930.4 | 427352.0000 | 3528.0000 | 0.4865 | 1.0033 | -1.0442 | 0.0039 |
| XM_004939528.1 | 101748775.0000 | 2338.0000 | 0.4417 | 0.9091 | -1.0415 | 0.0064 |
| NM_001277666.1 | 100858381.0000 | 994.0000 | 3.2137 | 6.5930 | -1.0367 | 0.0027 |
| XM_414478.4 | 416147.0000 | 1650.0000 | 5.0028 | 10.2419 | -1.0337 | 0.0001 |
| NM_204144.1 | 373943.0000 | 1321.0000 | 0.5535 | 1.1317 | -1.0318 | 0.0143 |
| NM_205101.1 | 395993.0000 | 2933.0000 | 1.9014 | 3.8829 | -1.0301 | 0.0003 |
| XM_004937769.1 | 101750812.0000 | 2805.0000 | 1.0866 | 2.2108 | -1.0247 | 0.0057 |
| XM_001236411.3 | 776920.0000 | 1227.0000 | 4.3750 | 8.8791 | -1.0211 | 0.0005 |
| NM_001030347.1 | 395342.0000 | 2116.0000 | 0.4686 | 0.9489 | -1.0178 | 0.0133 |
| XM_001235432.3 | 776306.0000 | 1948.0000 | 4.0685 | 8.2371 | -1.0176 | 0.0027 |
| XM_001234262.3 | 771920.0000 | 2239.0000 | 1.7346 | 3.5101 | -1.0169 | 0.0020 |
| XM_425293.4 | 427718.0000 | 2159.0000 | 0.3360 | 0.6781 | -1.0129 | 0.0144 |
| NM_204773.1 | 395546.0000 | 1691.0000 | 11.3640 | 22.8568 | -1.0082 | 0.0003 |
| XM_416896.4 | 418700.0000 | 1339.0000 | 2.8735 | 5.7781 | -1.0078 | 0.0018 |
| Novel002159.1 | Novel002159 | 5716.0000 | 0.7365 | 1.4800 | -1.0068 | 0.0014 |
| NM_001277370.1 | 417964.0000 | 2157.0000 | 13.4177 | 26.6130 | -0.9880 | 0.0001 |
| XM_416703.2 | 418495.0000 | 2081.0000 | 0.8764 | 1.7321 | -0.9829 | 0.0061 |
| XM_419701.4 | 421662.0000 | 3262.0000 | 1.9999 | 3.9510 | -0.9823 | 0.0115 |
| XM_004941610.1 | 100859610.0000 | 4196.0000 | 2.9949 | 5.9165 | -0.9823 | 0.0012 |
| XM_004946278.1 | 422080.0000 | 16117.0000 | 7.9175 | 15.6272 | -0.9809 | 0.0001 |
| NM_205344.1 | 396287.0000 | 900.0000 | 10.7972 | 21.2959 | -0.9799 | 0.0002 |
| NM_204335.1 | 374242.0000 | 1512.0000 | 6.2071 | 12.1953 | -0.9743 | 0.0001 |
| XM_421149.4 | 423225.0000 | 12233.0000 | 1.2513 | 2.4435 | -0.9656 | 0.0001 |
| NM_001031031.1 | 421199.0000 | 5390.0000 | 0.3305 | 0.6401 | -0.9537 | 0.0083 |
| XM_001232082.3 | 768922.0000 | 3626.0000 | 4.0629 | 7.8556 | -0.9512 | 0.0005 |
| XM_003643756.2 | 100859015.0000 | 711.0000 | 3.6336 | 7.0191 | -0.9499 | 0.0049 |
| NM_204171.1 | 373985.0000 | 4462.0000 | 1.6743 | 3.2270 | -0.9466 | 0.0002 |
| XM_416518.4 | 418295.0000 | 2702.0000 | 14.3908 | 27.7063 | -0.9451 | 0.0001 |
| NM_001245061.1 | 430189.0000 | 262.0000 | 170.7160 | 326.2200 | -0.9342 | 0.0001 |
| XM_003643209.2 | 100859012.0000 | 796.0000 | 2.6152 | 4.9525 | -0.9212 | 0.0098 |
| NM_001195401.1 | 428413.0000 | 882.0000 | 2.2034 | 4.1639 | -0.9182 | 0.0067 |
| XM_426693.4 | 429137.0000 | 3788.0000 | 14.5588 | 27.4686 | -0.9159 | 0.0001 |
| XM_004947259.1 | 419346.0000 | 7452.0000 | 1.4577 | 2.7499 | -0.9157 | 0.0128 |
| XM_423298.3 | 425545.0000 | 3089.0000 | 2.5923 | 4.8632 | -0.9077 | 0.0001 |
| NM_001030842.1 | 419112.0000 | 1377.0000 | 2.0881 | 3.9130 | -0.9061 | 0.0058 |
| XM_418206.4 | 420086.0000 | 2631.0000 | 0.9580 | 1.7918 | -0.9033 | 0.0072 |
| XM_424431.3 | 426821.0000 | 3472.0000 | 2.3458 | 4.3868 | -0.9031 | 0.0073 |
| NM_001031237.1 | 423896.0000 | 2923.0000 | 2.2244 | 4.1476 | -0.8988 | 0.0008 |
| XM_001234113.3 | 770787.0000 | 1808.0000 | 40.4511 | 75.2019 | -0.8946 | 0.0093 |
| NM_205128.1 | 396026.0000 | 1211.0000 | 1.9933 | 3.6942 | -0.8901 | 0.0063 |
| XM_003642244.2 | 100859100.0000 | 2097.0000 | 1.5015 | 2.7711 | -0.8840 | 0.0041 |
| NM_205080.1 | 395963.0000 | 2767.0000 | 6.8668 | 12.5672 | -0.8720 | 0.0001 |
| NM_001001613.1 | 414741.0000 | 1320.0000 | 253.5020 | 463.7850 | -0.8715 | 0.0001 |
| NM_204996.1 | 395845.0000 | 3574.0000 | 0.7454 | 1.3626 | -0.8703 | 0.0073 |
| XM_419840.4 | 421816.0000 | 2707.0000 | 82.0090 | 147.0620 | -0.8426 | 0.0001 |
| XM_413930.4 | 415560.0000 | 4486.0000 | 2.0308 | 3.6404 | -0.8421 | 0.0032 |
| NM_001162372.2 | 419083.0000 | 3084.0000 | 2.3510 | 4.2017 | -0.8377 | 0.0011 |
| NM_205163.1 | 396072.0000 | 3239.0000 | 1.3457 | 2.3911 | -0.8293 | 0.0037 |
| XM_415673.4 | 417421.0000 | 3084.0000 | 1.7417 | 3.0906 | -0.8274 | 0.0028 |
| XM_422896.4 | 425104.0000 | 760.0000 | 14.4778 | 25.6097 | -0.8228 | 0.0021 |
| NM_205495.1 | 396491.0000 | 498.0000 | 55.8948 | 98.2758 | -0.8141 | 0.0001 |
| Novel002165.1 | Novel002165 | 4687.0000 | 0.6721 | 1.1805 | -0.8127 | 0.0135 |
| NM_001030541.1 | 395429.0000 | 2655.0000 | 229.9140 | 403.7220 | -0.8123 | 0.0004 |
| NM_001030874.1 | 419424.0000 | 3060.0000 | 0.7960 | 1.3901 | -0.8043 | 0.0121 |
| XM_001235162.3 | 771952.0000 | 2730.0000 | 2.1290 | 3.7062 | -0.7998 | 0.0043 |
| NM_001135166.1 | 417051.0000 | 777.0000 | 9.5863 | 16.6660 | -0.7979 | 0.0033 |
| XM_001235378.3 | 772212.0000 | 3094.0000 | 1.0689 | 1.8566 | -0.7966 | 0.0109 |
| XM_420876.4 | 426948.0000 | 2284.0000 | 3.4703 | 6.0192 | -0.7945 | 0.0034 |
| XM_001234324.3 | 771003.0000 | 6597.0000 | 0.8142 | 1.4022 | -0.7842 | 0.0065 |
| NM_001127439.1 | 415487.0000 | 2170.0000 | 6.5350 | 11.2350 | -0.7817 | 0.0005 |
| XM_418929.4 | 420838.0000 | 2222.0000 | 5.4477 | 9.2811 | -0.7687 | 0.0007 |
| XM_003642893.2 | 420138.0000 | 6296.0000 | 5.5790 | 9.4962 | -0.7673 | 0.0001 |
| XM_003641899.2 | 415669.0000 | 490.0000 | 21.4997 | 36.5195 | -0.7644 | 0.0026 |
| NM_001012938.1 | 426315.0000 | 3832.0000 | 5.8460 | 9.9005 | -0.7601 | 0.0003 |
| NM_204535.4 | 395210.0000 | 1151.0000 | 6.7087 | 11.3543 | -0.7591 | 0.0103 |
| NM_001030883.1 | 419516.0000 | 3533.0000 | 2.7134 | 4.5634 | -0.7500 | 0.0013 |
| NM_001002856.1 | 403121.0000 | 489.0000 | 16.9771 | 28.4888 | -0.7468 | 0.0051 |
| XM_414712.4 | 408044.0000 | 6252.0000 | 11.4794 | 19.2351 | -0.7447 | 0.0001 |
| XM_004945666.1 | 693256.0000 | 624.0000 | 97.0053 | 162.1190 | -0.7409 | 0.0002 |
| NM_001031345.1 | 425657.0000 | 1485.0000 | 31.4114 | 52.3956 | -0.7382 | 0.0001 |
| XM_001235083.3 | 771862.0000 | 3756.0000 | 11.3200 | 18.7594 | -0.7287 | 0.0001 |
| NM_204635.1 | 395346.0000 | 3052.0000 | 2.6710 | 4.4246 | -0.7282 | 0.0033 |
| XM_419085.4 | 420999.0000 | 1938.0000 | 26.3188 | 43.5762 | -0.7274 | 0.0001 |
| NM_205416.1 | 396385.0000 | 1642.0000 | 12.0598 | 19.9365 | -0.7252 | 0.0004 |
| NM_205190.1 | 396107.0000 | 3339.0000 | 5.8845 | 9.7044 | -0.7217 | 0.0012 |
| NM_001277865.1 | 100858006.0000 | 682.0000 | 38.7817 | 63.7821 | -0.7178 | 0.0006 |
| XM_418617.4 | 420515.0000 | 4799.0000 | 3.2605 | 5.3614 | -0.7175 | 0.0010 |
| NM_204712.2 | 395451.0000 | 1533.0000 | 11.1635 | 18.2888 | -0.7122 | 0.0078 |
| NM_204839.2 | 395643.0000 | 1242.0000 | 14.9846 | 24.5475 | -0.7121 | 0.0008 |
| XM_424426.4 | 396331.0000 | 4402.0000 | 1.1524 | 1.8798 | -0.7059 | 0.0089 |
| NM_213572.1 | 404747.0000 | 2162.0000 | 2.0443 | 3.3228 | -0.7008 | 0.0144 |
| NM_204500.1 | 395161.0000 | 1956.0000 | 12.9605 | 21.0261 | -0.6981 | 0.0003 |
| XM_001231964.3 | 423263.0000 | 5198.0000 | 4.7388 | 7.6610 | -0.6930 | 0.0029 |
| XM_417496.4 | 395494.0000 | 3885.0000 | 1.8050 | 2.9175 | -0.6928 | 0.0064 |
| NM_001198600.1 | 395440.0000 | 1738.0000 | 24.1578 | 38.8886 | -0.6869 | 0.0003 |
| XM_414904.4 | 414781.0000 | 9692.0000 | 9.4850 | 15.2365 | -0.6838 | 0.0003 |
| NM_205231.1 | 396157.0000 | 3200.0000 | 2.8443 | 4.5536 | -0.6789 | 0.0048 |
| XM_416918.4 | 418719.0000 | 4451.0000 | 2.1128 | 3.3695 | -0.6734 | 0.0059 |
| NM_001037838.1 | 424540.0000 | 1136.0000 | 13.9884 | 22.3073 | -0.6733 | 0.0021 |
| NM_204861.1 | 395667.0000 | 3199.0000 | 7.4693 | 11.8790 | -0.6694 | 0.0006 |
| NM_205310.1 | 396248.0000 | 1369.0000 | 41.3099 | 65.6577 | -0.6685 | 0.0002 |
| NM_001193638.1 | 424185.0000 | 3888.0000 | 6.0079 | 9.5454 | -0.6680 | 0.0010 |
| XM_416333.4 | 418100.0000 | 2384.0000 | 5.6521 | 8.9697 | -0.6663 | 0.0027 |
| XM_414868.4 | 416567.0000 | 4810.0000 | 1.6961 | 2.6814 | -0.6608 | 0.0137 |
| XM_417710.3 | 419563.0000 | 3503.0000 | 12.2573 | 19.3379 | -0.6578 | 0.0010 |
| NM_001031609.2 | 431587.0000 | 2380.0000 | 134.2400 | 211.2890 | -0.6544 | 0.0011 |
| XM_417954.4 | 419818.0000 | 2964.0000 | 6.5705 | 10.3346 | -0.6534 | 0.0097 |
| NM_001011688.2 | 418638.0000 | 3455.0000 | 3.0844 | 4.8488 | -0.6527 | 0.0039 |
| XM_001232456.3 | 769222.0000 | 5055.0000 | 6.1879 | 9.7028 | -0.6489 | 0.0010 |
| NM_001006272.2 | 418865.0000 | 1936.0000 | 9.2987 | 14.5233 | -0.6433 | 0.0021 |
| NM_204493.1 | 395154.0000 | 2133.0000 | 12.4481 | 19.3754 | -0.6383 | 0.0019 |
| NM_001031279.1 | 424576.0000 | 3477.0000 | 2.3973 | 3.7154 | -0.6321 | 0.0092 |
| XM_416550.4 | 418327.0000 | 7612.0000 | 1.9803 | 3.0579 | -0.6268 | 0.0029 |
| NM_001031484.1 | 427947.0000 | 2679.0000 | 8.8956 | 13.6676 | -0.6196 | 0.0020 |
| XM_416206.4 | 417967.0000 | 2213.0000 | 14.2644 | 21.9081 | -0.6190 | 0.0017 |
| XM_422503.4 | 424670.0000 | 1919.0000 | 4.3747 | 6.7052 | -0.6161 | 0.0112 |
| NM_001004392.1 | 419243.0000 | 4918.0000 | 10.2925 | 15.7628 | -0.6149 | 0.0022 |
| NM_001012907.1 | 423826.0000 | 4032.0000 | 9.4791 | 14.5101 | -0.6142 | 0.0011 |
| XM_004939507.1 | 420728.0000 | 9724.0000 | 8.2191 | 12.5626 | -0.6121 | 0.0035 |
| XM_416660.4 | 418449.0000 | 6228.0000 | 19.6278 | 29.9943 | -0.6118 | 0.0009 |
| NM_001033643.1 | 419853.0000 | 1761.0000 | 9.2109 | 14.0283 | -0.6069 | 0.0058 |
| NM_001001604.1 | 414336.0000 | 1128.0000 | 11.5589 | 17.5761 | -0.6046 | 0.0106 |
| NM_204578.1 | 395266.0000 | 1944.0000 | 4.2876 | 6.5149 | -0.6036 | 0.0148 |
| NM_001201386.1 | 417943.0000 | 971.0000 | 22.2312 | 33.7468 | -0.6022 | 0.0035 |
| XM_004941244.1 | 428769.0000 | 6126.0000 | 1.7919 | 2.7140 | -0.5989 | 0.0117 |
| XM_003642278.2 | 427768.0000 | 2305.0000 | 6.7109 | 10.1489 | -0.5968 | 0.0059 |
| NM_001012914.1 | 424044.0000 | 3811.0000 | 32.6038 | 49.1922 | -0.5934 | 0.0050 |
| XM_417654.4 | 419504.0000 | 1224.0000 | 20.5612 | 30.9923 | -0.5920 | 0.0035 |
| XM_424455.4 | 426846.0000 | 1632.0000 | 7.5153 | 11.2673 | -0.5843 | 0.0114 |
| XM_003643759.2 | 429035.0000 | 927.0000 | 121.2290 | 180.8240 | -0.5769 | 0.0016 |
| NM_204954.1 | 395797.0000 | 2343.0000 | 28.9742 | 42.9485 | -0.5678 | 0.0031 |
| XM_004945551.1 | 396405.0000 | 4680.0000 | 3.9070 | 5.7766 | -0.5642 | 0.0115 |
| NM_001012596.1 | 424451.0000 | 4039.0000 | 22.7220 | 33.5926 | -0.5641 | 0.0027 |
| NM_001005346.1 | 419088.0000 | 2606.0000 | 6.1708 | 9.1205 | -0.5637 | 0.0144 |
| XM_001232431.3 | 769203.0000 | 2008.0000 | 11.7516 | 17.3523 | -0.5623 | 0.0069 |
| NM_001081703.1 | 427896.0000 | 743.0000 | 34.9460 | 51.5837 | -0.5618 | 0.0062 |
| XM_003641478.2 | 423781.0000 | 10475.0000 | 2.6214 | 3.8631 | -0.5594 | 0.0050 |
| NM_204149.1 | 373952.0000 | 1846.0000 | 23.3418 | 34.3857 | -0.5589 | 0.0031 |
| NM_001006191.1 | 416884.0000 | 2911.0000 | 7.1931 | 10.5850 | -0.5573 | 0.0061 |
| NM_204860.2 | 395666.0000 | 4376.0000 | 12.2565 | 17.9870 | -0.5534 | 0.0073 |
| NM_205155.2 | 396061.0000 | 10634.0000 | 11.9215 | 17.4879 | -0.5528 | 0.0037 |
| NM_001195557.1 | 417981.0000 | 2810.0000 | 8.2463 | 12.0926 | -0.5523 | 0.0055 |
| NM_001193639.2 | 424186.0000 | 3113.0000 | 29.7757 | 43.6371 | -0.5514 | 0.0050 |
| NM_001244593.1 | 420588.0000 | 4690.0000 | 5.9391 | 8.6985 | -0.5505 | 0.0071 |
| NM_204787.1 | 395565.0000 | 12300.0000 | 12.3676 | 18.0983 | -0.5493 | 0.0041 |
| NM_001163232.2 | 427638.0000 | 1026.0000 | 482.8650 | 706.2040 | -0.5485 | 0.0065 |
| XM_417886.3 | 419747.0000 | 4102.0000 | 9.0838 | 13.2664 | -0.5464 | 0.0042 |
| XM_001231675.3 | 768904.0000 | 1956.0000 | 40.3011 | 58.7953 | -0.5449 | 0.0066 |
| XM_425982.4 | 428421.0000 | 6149.0000 | 5.6927 | 8.2898 | -0.5422 | 0.0036 |
| NM_204891.1 | 395707.0000 | 3841.0000 | 6.0163 | 8.7471 | -0.5399 | 0.0061 |
| NM_001277702.1 | 416156.0000 | 2502.0000 | 15.0505 | 21.7225 | -0.5294 | 0.0063 |
| Novel002568.1 | Novel002568 | 2907.0000 | 21.2484 | 30.6576 | -0.5289 | 0.0101 |
| XM_426077.4 | 428523.0000 | 5659.0000 | 2.1764 | 3.1329 | -0.5256 | 0.0153 |
| NM_001277738.1 | 418637.0000 | 2311.0000 | 15.7473 | 22.6239 | -0.5227 | 0.0090 |
| XM_422113.4 | 424266.0000 | 10592.0000 | 12.1243 | 17.4097 | -0.5220 | 0.0057 |
| XM_004937371.1 | 396174.0000 | 12146.0000 | 6.5096 | 9.2973 | -0.5142 | 0.0072 |
| XM_416662.4 | 418451.0000 | 6248.0000 | 15.9307 | 22.7039 | -0.5111 | 0.0063 |
| NM_001199502.1 | 420357.0000 | 2385.0000 | 46.5829 | 66.2176 | -0.5074 | 0.0050 |
| XM_414209.4 | 415849.0000 | 9660.0000 | 3.9857 | 5.6593 | -0.5058 | 0.0136 |
| XM_001234659.3 | 424803.0000 | 7146.0000 | 7.5184 | 10.6754 | -0.5058 | 0.0059 |
| NM_204255.1 | 374105.0000 | 4403.0000 | 8.8685 | 12.5842 | -0.5049 | 0.0080 |
| NM_204971.1 | 395818.0000 | 1186.0000 | 25.2749 | 35.7711 | -0.5011 | 0.0111 |
| NM_205351.1 | 396297.0000 | 1368.0000 | 102.9360 | 145.6810 | -0.5011 | 0.0080 |
| XM_003640331.2 | 425522.0000 | 8767.0000 | 6.3871 | 9.0151 | -0.4972 | 0.0071 |
| NM_205251.1 | 396181.0000 | 3346.0000 | 9.0516 | 12.7722 | -0.4968 | 0.0132 |
| XM_003641501.2 | 100857572.0000 | 1441.0000 | 67.9020 | 95.5582 | -0.4929 | 0.0074 |
| XM_004941563.1 | 101748745.0000 | 1637.0000 | 17.8806 | 25.1424 | -0.4917 | 0.0131 |
| XM_001231970.3 | 769847.0000 | 1491.0000 | 19.8091 | 27.8111 | -0.4895 | 0.0127 |
| NM_204290.1 | 374165.0000 | 474.0000 | 122.1970 | 171.2760 | -0.4871 | 0.0126 |
| NM_001008440.1 | 418852.0000 | 3309.0000 | 22.3133 | 31.2617 | -0.4865 | 0.0106 |
| NM_001031012.1 | 421037.0000 | 1341.0000 | 22.5746 | 31.5947 | -0.4850 | 0.0139 |
| NM_205298.2 | 396235.0000 | 1762.0000 | 14.6075 | 20.3992 | -0.4818 | 0.0141 |
| XM_417299.4 | 419111.0000 | 5292.0000 | 33.0986 | 46.0706 | -0.4771 | 0.0113 |
| NM_204666.1 | 395386.0000 | 2184.0000 | 119.6670 | 166.5160 | -0.4766 | 0.0124 |
| NM_001160428.2 | 423122.0000 | 2142.0000 | 19.8612 | 27.5218 | -0.4706 | 0.0130 |
| XM_004938929.1 | 419027.0000 | 9343.0000 | 4.3711 | 6.0559 | -0.4703 | 0.0135 |
| NM_001277853.1 | 100857115.0000 | 865.0000 | 54.0702 | 74.7273 | -0.4668 | 0.0148 |
| XM_416910.4 | 418711.0000 | 2150.0000 | 39.6851 | 29.2165 | 0.4418 | 0.0149 |
| NM_205205.1 | 396125.0000 | 2207.0000 | 51.2587 | 37.6475 | 0.4452 | 0.0123 |
| XM_424265.4 | 426636.0000 | 2310.0000 | 35.9820 | 26.4072 | 0.4463 | 0.0150 |
| XM_420691.4 | 422736.0000 | 3479.0000 | 23.5910 | 17.2878 | 0.4485 | 0.0133 |
| NM_204853.1 | 395659.0000 | 1437.0000 | 80.4399 | 58.8316 | 0.4513 | 0.0142 |
| NM_001030584.1 | 415906.0000 | 491.0000 | 206.1330 | 150.6270 | 0.4526 | 0.0152 |
| XM_427026.4 | 429470.0000 | 4269.0000 | 15.9755 | 11.6475 | 0.4558 | 0.0139 |
| XM_003643809.2 | 100859241.0000 | 961.0000 | 191.4190 | 139.5570 | 0.4559 | 0.0139 |
| NM_001005798.1 | 404774.0000 | 4014.0000 | 22.8447 | 16.6429 | 0.4569 | 0.0126 |
| NM_001278156.1 | 421156.0000 | 1423.0000 | 91.8436 | 66.8628 | 0.4580 | 0.0110 |
| NM_001030735.1 | 417783.0000 | 3213.0000 | 62.2376 | 45.2308 | 0.4605 | 0.0131 |
| XM_422828.4 | 425026.0000 | 2387.0000 | 59.5336 | 43.1471 | 0.4644 | 0.0137 |
| Novel000636.1 | Novel000636 | 30419.0000 | 2.4837 | 1.7979 | 0.4662 | 0.0132 |
| NM_213575.1 | 404752.0000 | 1083.0000 | 100.5480 | 72.6419 | 0.4690 | 0.0116 |
| XM_417563.3 | 419401.0000 | 4227.0000 | 47.6237 | 34.3948 | 0.4695 | 0.0122 |
| XM_418544.4 | 420439.0000 | 2242.0000 | 184.9300 | 133.1110 | 0.4744 | 0.0126 |
| NM_001113291.1 | 431066.0000 | 1528.0000 | 138.5610 | 99.4139 | 0.4790 | 0.0083 |
| NM_001190925.1 | 768717.0000 | 6671.0000 | 24.5384 | 17.5358 | 0.4847 | 0.0083 |
| NM_213580.2 | 404773.0000 | 548.0000 | 268.7410 | 191.5580 | 0.4884 | 0.0114 |
| XM_413725.3 | 415339.0000 | 5308.0000 | 12.4087 | 8.8403 | 0.4892 | 0.0072 |
| XM_424543.3 | 426932.0000 | 3186.0000 | 32.5173 | 23.1502 | 0.4902 | 0.0099 |
| NM_205454.1 | 396438.0000 | 1796.0000 | 121.3870 | 86.3945 | 0.4906 | 0.0080 |
| NM_001168009.1 | 427466.0000 | 1398.0000 | 192.4360 | 136.9360 | 0.4909 | 0.0097 |
| XM_429181.4 | 431629.0000 | 556.0000 | 596.2310 | 423.6050 | 0.4932 | 0.0070 |
| NM_001199569.1 | 423023.0000 | 2169.0000 | 21.3852 | 15.1864 | 0.4938 | 0.0120 |
| NM_001030889.1 | 419557.0000 | 402.0000 | 488.8000 | 346.9340 | 0.4946 | 0.0077 |
| NM_001031011.1 | 421035.0000 | 2229.0000 | 19.3782 | 13.7270 | 0.4974 | 0.0108 |
| NM_001190802.1 | 770135.0000 | 464.0000 | 930.5500 | 658.7460 | 0.4984 | 0.0086 |
| XM_003642832.2 | 772158.0000 | 821.0000 | 447.4750 | 316.1710 | 0.5011 | 0.0076 |
| NM_001031217.1 | 423621.0000 | 2304.0000 | 275.7310 | 194.5160 | 0.5034 | 0.0148 |
| XM_425654.4 | 428096.0000 | 3727.0000 | 13.8748 | 9.7867 | 0.5036 | 0.0081 |
| XM_426809.4 | 429253.0000 | 1369.0000 | 51.2059 | 36.0703 | 0.5055 | 0.0069 |
| XM_003642852.2 | 100858798.0000 | 1301.0000 | 139.7780 | 98.4462 | 0.5057 | 0.0060 |
| XM_424500.4 | 426894.0000 | 2404.0000 | 176.0760 | 123.8850 | 0.5072 | 0.0090 |
| XM_001231897.2 | 427467.0000 | 4098.0000 | 12.8585 | 9.0458 | 0.5074 | 0.0106 |
| NM_001031612.2 | 431669.0000 | 2874.0000 | 15.7335 | 11.0430 | 0.5107 | 0.0081 |
| XM_417822.4 | 419675.0000 | 3051.0000 | 25.3384 | 17.7738 | 0.5116 | 0.0120 |
| XM_003642957.2 | 416366.0000 | 2499.0000 | 16.0109 | 11.2309 | 0.5116 | 0.0099 |
| NM_001044678.1 | 693255.0000 | 536.0000 | 66.1852 | 46.3018 | 0.5154 | 0.0151 |
| XM_422971.4 | 425184.0000 | 669.0000 | 53.7147 | 37.5349 | 0.5171 | 0.0132 |
| XM_001234759.3 | 771478.0000 | 524.0000 | 399.4730 | 278.3170 | 0.5214 | 0.0042 |
| NM_001277811.1 | 418978.0000 | 4661.0000 | 31.7079 | 22.0819 | 0.5220 | 0.0042 |
| XM_418200.4 | 420080.0000 | 1557.0000 | 28.7290 | 19.9875 | 0.5234 | 0.0068 |
| XM_004939624.1 | 420827.0000 | 3202.0000 | 11.7388 | 8.1070 | 0.5341 | 0.0121 |
| NM_001031420.1 | 427237.0000 | 2085.0000 | 99.2267 | 68.3803 | 0.5371 | 0.0049 |
| NM_001004768.1 | 448832.0000 | 2095.0000 | 22.1616 | 15.2513 | 0.5391 | 0.0053 |
| XM_001235075.3 | 771852.0000 | 513.0000 | 105.3090 | 72.3631 | 0.5413 | 0.0057 |
| XM_424624.4 | 427022.0000 | 2441.0000 | 77.7329 | 53.3236 | 0.5438 | 0.0035 |
| NM_001199910.1 | 428372.0000 | 4008.0000 | 77.8893 | 53.4299 | 0.5438 | 0.0041 |
| XM_419897.4 | 421880.0000 | 1104.0000 | 227.0280 | 155.4940 | 0.5460 | 0.0031 |
| NM_204274.1 | 374135.0000 | 2273.0000 | 54.3444 | 37.2023 | 0.5467 | 0.0045 |
| XM_419574.4 | 421533.0000 | 4308.0000 | 18.7139 | 12.8067 | 0.5472 | 0.0040 |
| XM_418258.4 | 420141.0000 | 1030.0000 | 109.9780 | 75.1077 | 0.5502 | 0.0035 |
| NM_001278149.1 | 771364.0000 | 1165.0000 | 17.0364 | 11.6132 | 0.5529 | 0.0115 |
| NM_001006557.3 | 425041.0000 | 1465.0000 | 89.1914 | 60.7726 | 0.5535 | 0.0023 |
| XM_004940673.1 | 422238.0000 | 1351.0000 | 207.8450 | 141.5860 | 0.5538 | 0.0110 |
| NM_001009928.1 | 404779.0000 | 1110.0000 | 19.0217 | 12.9501 | 0.5547 | 0.0125 |
| XM_416687.4 | 418479.0000 | 4185.0000 | 27.8636 | 18.9553 | 0.5558 | 0.0023 |
| NM_205256.1 | 396191.0000 | 2462.0000 | 46.7345 | 31.6095 | 0.5641 | 0.0048 |
| NM_001001764.1 | 414873.0000 | 1281.0000 | 730.7910 | 491.8960 | 0.5711 | 0.0144 |
| XM_004947710.1 | 101748332.0000 | 633.0000 | 56.2776 | 37.7650 | 0.5755 | 0.0058 |
| XM_001231253.3 | 768374.0000 | 3200.0000 | 169.1540 | 113.2600 | 0.5787 | 0.0054 |
| XM_004947671.1 | 419547.0000 | 4402.0000 | 24.9014 | 16.6613 | 0.5797 | 0.0083 |
| NM_001030695.1 | 417303.0000 | 3387.0000 | 6.2825 | 4.1991 | 0.5813 | 0.0069 |
| NM_001159698.1 | 418917.0000 | 3334.0000 | 14.2890 | 9.5470 | 0.5818 | 0.0027 |
| XM_004949341.1 | 431620.0000 | 1593.0000 | 13.2392 | 8.8435 | 0.5821 | 0.0084 |
| XM_003642781.2 | 427072.0000 | 1800.0000 | 23.9407 | 15.9840 | 0.5828 | 0.0109 |
| XM_001234185.3 | 770867.0000 | 1595.0000 | 104.1380 | 69.4827 | 0.5838 | 0.0031 |
| XM_424461.3 | 426853.0000 | 6077.0000 | 17.3728 | 11.5837 | 0.5847 | 0.0022 |
| XM_417598.4 | 419439.0000 | 1891.0000 | 32.5940 | 21.7027 | 0.5867 | 0.0028 |
| NM_204967.1 | 395814.0000 | 2610.0000 | 10.4563 | 6.9598 | 0.5873 | 0.0057 |
| XM_004948033.1 | 395766.0000 | 2398.0000 | 40.1384 | 26.6070 | 0.5932 | 0.0021 |
| XM_004944510.1 | 100858928.0000 | 4025.0000 | 4.5151 | 2.9925 | 0.5934 | 0.0059 |
| NM_204155.1 | 373960.0000 | 1461.0000 | 154.3520 | 102.0340 | 0.5972 | 0.0013 |
| XM_420239.4 | 422253.0000 | 6753.0000 | 35.8917 | 23.7090 | 0.5982 | 0.0013 |
| XM_415293.4 | 417002.0000 | 4292.0000 | 12.1474 | 8.0183 | 0.5993 | 0.0053 |
| Novel001658.1 | Novel001658 | 443.0000 | 157.0740 | 103.5800 | 0.6007 | 0.0138 |
| NM_001012595.2 | 424396.0000 | 3088.0000 | 48.2129 | 31.7932 | 0.6007 | 0.0009 |
| NM_204511.1 | 395182.0000 | 1220.0000 | 50.0932 | 32.9619 | 0.6038 | 0.0021 |
| XM_417029.4 | 418833.0000 | 1783.0000 | 61.4847 | 40.4572 | 0.6038 | 0.0014 |
| NM_001012911.1 | 423888.0000 | 2331.0000 | 46.7389 | 30.6662 | 0.6080 | 0.0009 |
| NM_001012886.1 | 422344.0000 | 3503.0000 | 18.2181 | 11.9293 | 0.6109 | 0.0017 |
| XM_424463.4 | 426854.0000 | 1194.0000 | 156.3280 | 102.0640 | 0.6151 | 0.0025 |
| NM_204745.1 | 395504.0000 | 2090.0000 | 363.7750 | 236.6100 | 0.6205 | 0.0073 |
| XM_426713.4 | 429158.0000 | 5314.0000 | 96.7327 | 62.7519 | 0.6243 | 0.0021 |
| NM_001278084.1 | 418549.0000 | 7211.0000 | 33.2576 | 21.5657 | 0.6249 | 0.0009 |
| XM_004945322.1 | 416642.0000 | 3649.0000 | 22.0773 | 14.2747 | 0.6291 | 0.0033 |
| XM_421126.4 | 423202.0000 | 2848.0000 | 5.0395 | 3.2582 | 0.6292 | 0.0088 |
| NM_205445.1 | 396427.0000 | 3079.0000 | 5.9244 | 3.8095 | 0.6371 | 0.0046 |
| XM_003643544.2 | 100857880.0000 | 681.0000 | 31.9181 | 20.4278 | 0.6438 | 0.0052 |
| XM_001235152.3 | 771943.0000 | 1284.0000 | 12.1200 | 7.7508 | 0.6450 | 0.0086 |
| XM_003642600.2 | 769528.0000 | 8366.0000 | 6.4972 | 4.1546 | 0.6451 | 0.0027 |
| NM_204573.1 | 395260.0000 | 268.0000 | 129.5260 | 82.6044 | 0.6490 | 0.0152 |
| Novel001540.1 | Novel001540 | 21837.0000 | 0.5029 | 0.3196 | 0.6542 | 0.0093 |
| XM_004940811.1 | 422348.0000 | 7697.0000 | 22.8969 | 14.5494 | 0.6542 | 0.0017 |
| NM_204568.1 | 395255.0000 | 1622.0000 | 32.4057 | 20.4965 | 0.6609 | 0.0004 |
| XM_416112.4 | 417870.0000 | 3906.0000 | 3.8104 | 2.4094 | 0.6613 | 0.0061 |
| NM_204107.1 | 373896.0000 | 1542.0000 | 288.0970 | 181.3950 | 0.6674 | 0.0005 |
| XM_001235058.3 | 771832.0000 | 2076.0000 | 9.5051 | 5.9758 | 0.6696 | 0.0031 |
| XM_003641316.2 | 423031.0000 | 8979.0000 | 31.3863 | 19.6900 | 0.6727 | 0.0040 |
| NM_001031605.1 | 431449.0000 | 3118.0000 | 39.9274 | 24.9710 | 0.6771 | 0.0001 |
| XM_421587.4 | 423708.0000 | 994.0000 | 95.2314 | 59.3363 | 0.6825 | 0.0014 |
| NM_001044651.1 | 422365.0000 | 2067.0000 | 457.1300 | 284.6620 | 0.6834 | 0.0019 |
| NM_205320.1 | 396260.0000 | 585.0000 | 206.0140 | 128.1510 | 0.6849 | 0.0003 |
| NM_205476.1 | 396468.0000 | 337.0000 | 78.4232 | 48.7819 | 0.6849 | 0.0075 |
| XM_001234250.2 | 770939.0000 | 2274.0000 | 18.2654 | 11.3417 | 0.6875 | 0.0009 |
| XM_003641788.2 | 769039.0000 | 668.0000 | 26.8354 | 16.6306 | 0.6903 | 0.0040 |
| XM_004937174.1 | 431664.0000 | 2705.0000 | 3.4296 | 2.1244 | 0.6910 | 0.0114 |
| XM_001234466.3 | 771172.0000 | 4028.0000 | 13.1717 | 8.1379 | 0.6947 | 0.0006 |
| XM_417914.4 | 419776.0000 | 2016.0000 | 5.2205 | 3.2060 | 0.7034 | 0.0098 |
| XM_424774.4 | 427187.0000 | 3768.0000 | 45.7594 | 28.0539 | 0.7059 | 0.0002 |
| XM_427433.4 | 429878.0000 | 2842.0000 | 9.8424 | 6.0319 | 0.7064 | 0.0020 |
| XM_003640984.2 | 100859813.0000 | 12488.0000 | 0.7940 | 0.4841 | 0.7140 | 0.0059 |
| NM_001277461.1 | 415462.0000 | 1341.0000 | 87.2304 | 53.1726 | 0.7141 | 0.0002 |
| XM_424718.4 | 427125.0000 | 898.0000 | 64.5058 | 39.2064 | 0.7183 | 0.0001 |
| XM_417040.4 | 418845.0000 | 2012.0000 | 153.2260 | 92.8103 | 0.7233 | 0.0001 |
| NM_001110177.1 | 421775.0000 | 2622.0000 | 3.2235 | 1.9498 | 0.7253 | 0.0089 |
| XM_004934549.1 | 427984.0000 | 2255.0000 | 6.1527 | 3.7102 | 0.7297 | 0.0043 |
| XM_004943816.1 | 415455.0000 | 3740.0000 | 12.8047 | 7.6928 | 0.7351 | 0.0066 |
| XM_004937947.1 | 772197.0000 | 2682.0000 | 15.6072 | 9.3486 | 0.7394 | 0.0031 |
| XM_422985.4 | 425201.0000 | 1427.0000 | 4.9196 | 2.9411 | 0.7422 | 0.0112 |
| NM_205021.1 | 395875.0000 | 9950.0000 | 16.5161 | 9.8461 | 0.7462 | 0.0001 |
| XM_424964.3 | 427391.0000 | 1558.0000 | 17.1487 | 10.2230 | 0.7463 | 0.0002 |
| XM_001232838.3 | 770392.0000 | 1984.0000 | 5.5206 | 3.2869 | 0.7481 | 0.0057 |
| NM_001105666.1 | 100049619.0000 | 4816.0000 | 3.2145 | 1.9052 | 0.7547 | 0.0011 |
| NM_204263.1 | 374117.0000 | 1716.0000 | 28.2487 | 16.7164 | 0.7569 | 0.0003 |
| XM_418326.4 | 420216.0000 | 4075.0000 | 9.5187 | 5.6252 | 0.7589 | 0.0004 |
| NM_204457.1 | 395108.0000 | 1102.0000 | 9.1226 | 5.3694 | 0.7647 | 0.0149 |
| NM_204467.1 | 395121.0000 | 1532.0000 | 13.7414 | 8.0375 | 0.7737 | 0.0006 |
| XM_419778.4 | 421746.0000 | 1531.0000 | 112.2210 | 65.5033 | 0.7767 | 0.0001 |
| NM_205288.1 | 396225.0000 | 3527.0000 | 9.4496 | 5.5157 | 0.7767 | 0.0003 |
| XM_004937471.1 | 418247.0000 | 4396.0000 | 47.9396 | 27.8148 | 0.7854 | 0.0001 |
| Novel002179.1 | Novel002179 | 18283.0000 | 3.8727 | 2.2445 | 0.7870 | 0.0056 |
| XM_003642222.2 | 416933.0000 | 664.0000 | 123.0830 | 71.2192 | 0.7893 | 0.0001 |
| XM_004942838.1 | 395906.0000 | 1516.0000 | 1534.4100 | 885.1400 | 0.7937 | 0.0106 |
| XM_003640811.2 | 100858989.0000 | 2699.0000 | 10.7747 | 6.2113 | 0.7947 | 0.0067 |
| NM_001177739.1 | 771738.0000 | 571.0000 | 117.7210 | 67.6986 | 0.7982 | 0.0001 |
| NM_204587.1 | 395279.0000 | 5927.0000 | 2.3646 | 1.3580 | 0.8001 | 0.0059 |
| XM_417600.4 | 419442.0000 | 1404.0000 | 16.6122 | 9.4823 | 0.8089 | 0.0003 |
| XM_004938556.1 | 769178.0000 | 1380.0000 | 55.3267 | 31.3820 | 0.8180 | 0.0002 |
| XM_413700.4 | 415313.0000 | 1769.0000 | 7.6826 | 4.3554 | 0.8188 | 0.0014 |
| XM_420498.4 | 422535.0000 | 3725.0000 | 3.9318 | 2.2228 | 0.8228 | 0.0013 |
| NM_204818.2 | 395612.0000 | 842.0000 | 10.0286 | 5.6482 | 0.8283 | 0.0047 |
| XM_422171.4 | 424326.0000 | 3492.0000 | 1.0514 | 0.5900 | 0.8334 | 0.0144 |
| NM_001031317.1 | 424940.0000 | 1542.0000 | 13.1899 | 7.3589 | 0.8419 | 0.0006 |
| XM_004945953.1 | 417147.0000 | 5316.0000 | 12.5276 | 6.9666 | 0.8466 | 0.0024 |
| NM_001199486.1 | 422979.0000 | 1208.0000 | 4.0011 | 2.2232 | 0.8478 | 0.0114 |
| NM_204736.1 | 395488.0000 | 1765.0000 | 8.5074 | 4.7235 | 0.8489 | 0.0006 |
| XM_418978.4 | 420893.0000 | 7483.0000 | 133.2650 | 73.6646 | 0.8553 | 0.0011 |
| NM_001177575.1 | 420401.0000 | 1760.0000 | 1.6422 | 0.9048 | 0.8600 | 0.0151 |
| NM_001039264.2 | 416826.0000 | 1085.0000 | 72.8654 | 40.0762 | 0.8625 | 0.0001 |
| NM_204705.1 | 395444.0000 | 1752.0000 | 3.0273 | 1.6480 | 0.8773 | 0.0059 |
| XM_004947675.1 | 419548.0000 | 2886.0000 | 4.4092 | 2.3771 | 0.8913 | 0.0091 |
| Novel001230.1 | Novel001230 | 918.0000 | 29.1467 | 15.6913 | 0.8934 | 0.0011 |
| XM_001233743.3 | 770405.0000 | 1303.0000 | 10.1559 | 5.4356 | 0.9018 | 0.0038 |
| NM_001276362.1 | 418125.0000 | 6878.0000 | 4.2379 | 2.2579 | 0.9084 | 0.0001 |
| NM_001135679.1 | 417616.0000 | 1833.0000 | 2.1020 | 1.1192 | 0.9093 | 0.0133 |
| NM_001277696.1 | 414890.0000 | 1693.0000 | 2.2550 | 1.1982 | 0.9123 | 0.0077 |
| XM_001232282.3 | 769085.0000 | 4343.0000 | 3.1202 | 1.6550 | 0.9148 | 0.0010 |
| Novel002850.1 | Novel002850 | 5312.0000 | 0.6615 | 0.3504 | 0.9168 | 0.0081 |
| XM_004944976.1 | 416304.0000 | 4587.0000 | 0.5159 | 0.2732 | 0.9173 | 0.0154 |
| XM_416631.4 | 418417.0000 | 1174.0000 | 14.5565 | 7.6762 | 0.9232 | 0.0040 |
| NM_204218.1 | 374054.0000 | 1887.0000 | 4.7076 | 2.4765 | 0.9267 | 0.0014 |
| XM_003643158.2 | 100858580.0000 | 341.0000 | 13.0806 | 6.8559 | 0.9320 | 0.0151 |
| XM_004947496.1 | 768390.0000 | 1732.0000 | 6.1138 | 3.1961 | 0.9357 | 0.0055 |
| XM_414534.4 | 416208.0000 | 2397.0000 | 11.5771 | 6.0397 | 0.9387 | 0.0001 |
| NM_001030956.1 | 420343.0000 | 4562.0000 | 209.1650 | 109.0920 | 0.9391 | 0.0001 |
| XM_428114.4 | 430557.0000 | 8219.0000 | 2.9134 | 1.5121 | 0.9461 | 0.0001 |
| XM_004949384.1 | 431569.0000 | 5309.0000 | 28.0491 | 14.2636 | 0.9756 | 0.0001 |
| XM_003642981.2 | 100857191.0000 | 1726.0000 | 7.9739 | 4.0099 | 0.9917 | 0.0003 |
| XM_421810.4 | 423951.0000 | 5132.0000 | 2.0136 | 1.0124 | 0.9920 | 0.0005 |
| XM_422246.4 | 424402.0000 | 2842.0000 | 10.0334 | 5.0441 | 0.9922 | 0.0001 |
| XM_004945664.1 | 101747935.0000 | 5253.0000 | 1.1545 | 0.5773 | 0.9999 | 0.0013 |
| XM_417682.3 | 419530.0000 | 1067.0000 | 6.5044 | 3.2464 | 1.0026 | 0.0017 |
| NM_204567.1 | 395254.0000 | 544.0000 | 103.7600 | 51.6940 | 1.0052 | 0.0001 |
| NM_001006368.1 | 420706.0000 | 2423.0000 | 5.3285 | 2.6451 | 1.0104 | 0.0002 |
| XM_003643019.2 | 770238.0000 | 4216.0000 | 64.2573 | 31.7706 | 1.0162 | 0.0001 |
| NM_001252130.1 | 768482.0000 | 1433.0000 | 11.4560 | 5.6141 | 1.0290 | 0.0001 |
| XM_004949789.1 | 101749549.0000 | 789.0000 | 3.3442 | 1.6178 | 1.0476 | 0.0086 |
| NM_001139439.1 | 420315.0000 | 5316.0000 | 2.4321 | 1.1660 | 1.0606 | 0.0001 |
| XM_419752.4 | 421715.0000 | 1891.0000 | 1.6844 | 0.8058 | 1.0637 | 0.0064 |
| XM_003641175.2 | 100857543.0000 | 2720.0000 | 0.9573 | 0.4568 | 1.0675 | 0.0058 |
| NM_001277611.1 | 429967.0000 | 822.0000 | 1.9139 | 0.9028 | 1.0841 | 0.0126 |
| XM_003642241.2 | 768349.0000 | 3740.0000 | 1.1709 | 0.5498 | 1.0907 | 0.0016 |
| XM_417393.4 | 419216.0000 | 3253.0000 | 1.9652 | 0.9073 | 1.1151 | 0.0041 |
| NM_001198927.1 | 424862.0000 | 2338.0000 | 5.5859 | 2.5595 | 1.1260 | 0.0001 |
| NM_001199458.1 | 417708.0000 | 1906.0000 | 1.5718 | 0.7162 | 1.1339 | 0.0027 |
| XM_419622.3 | 421584.0000 | 1809.0000 | 0.7881 | 0.3582 | 1.1378 | 0.0138 |
| NM_205159.1 | 396067.0000 | 909.0000 | 20.4601 | 9.1757 | 1.1569 | 0.0001 |
| NM_001199656.1 | 423209.0000 | 1945.0000 | 198.0660 | 88.7776 | 1.1577 | 0.0001 |
| XM_415036.4 | 416740.0000 | 2610.0000 | 4.2696 | 1.9121 | 1.1589 | 0.0002 |
| NM_001113289.1 | 423196.0000 | 429.0000 | 4.2663 | 1.8893 | 1.1752 | 0.0116 |
| XM_429539.4 | 417973.0000 | 1517.0000 | 8.5460 | 3.7653 | 1.1825 | 0.0001 |
| XM_003642426.2 | 100858782.0000 | 2298.0000 | 50.9231 | 22.4276 | 1.1830 | 0.0077 |
| XM_417126.4 | 418932.0000 | 986.0000 | 4.0649 | 1.7529 | 1.2134 | 0.0015 |
| NM_001097526.1 | 417506.0000 | 973.0000 | 22.5872 | 9.6673 | 1.2243 | 0.0001 |
| NM_205345.2 | 396289.0000 | 2199.0000 | 0.6739 | 0.2883 | 1.2248 | 0.0091 |
| XM_414882.4 | 416581.0000 | 1701.0000 | 1.0716 | 0.4575 | 1.2278 | 0.0033 |
| NM_001031810.1 | 417035.0000 | 1790.0000 | 3.3947 | 1.4482 | 1.2291 | 0.0153 |
| XM_419553.4 | 421508.0000 | 15756.0000 | 0.5893 | 0.2496 | 1.2393 | 0.0005 |
| NM_204897.1 | 395715.0000 | 2077.0000 | 1.0003 | 0.4217 | 1.2461 | 0.0020 |
| XM_004948913.1 | 771165.0000 | 2148.0000 | 37.3442 | 15.6804 | 1.2519 | 0.0001 |
| XM_004934886.1 | 419814.0000 | 4115.0000 | 28.3413 | 11.8654 | 1.2562 | 0.0062 |
| XM_004949251.1 | 425241.0000 | 1667.0000 | 6.1788 | 2.5801 | 1.2599 | 0.0007 |
| XM_004940307.1 | 421758.0000 | 1651.0000 | 2.1467 | 0.8933 | 1.2649 | 0.0046 |
| XM_004935409.1 | 101751239.0000 | 1109.0000 | 3.8667 | 1.6068 | 1.2669 | 0.0005 |
| Novel002016.1 | Novel002016 | 15720.0000 | 2.8347 | 1.1753 | 1.2702 | 0.0023 |
| NM_001185051.1 | 422019.0000 | 600.0000 | 3.2037 | 1.3257 | 1.2730 | 0.0065 |
| XM_003641370.2 | 423427.0000 | 1878.0000 | 2.1330 | 0.8825 | 1.2732 | 0.0087 |
| NM_001270816.1 | 428083.0000 | 678.0000 | 8.3125 | 3.4363 | 1.2744 | 0.0004 |
| XM_001235203.3 | 772003.0000 | 1565.0000 | 1.1487 | 0.4732 | 1.2795 | 0.0038 |
| Novel000034.1 | Novel000034 | 516.0000 | 7.5938 | 3.1215 | 1.2826 | 0.0013 |
| XM_001234162.3 | 770839.0000 | 793.0000 | 18.0372 | 7.2317 | 1.3186 | 0.0001 |
| NM_001100289.1 | 427553.0000 | 3652.0000 | 3.5203 | 1.3897 | 1.3410 | 0.0001 |
| XM_004949074.1 | 427227.0000 | 6942.0000 | 19.4694 | 7.5870 | 1.3596 | 0.0014 |
| NM_205223.1 | 396146.0000 | 894.0000 | 38.1785 | 14.7831 | 1.3688 | 0.0001 |
| XM_004937556.1 | 101750146.0000 | 2272.0000 | 8.3307 | 3.2035 | 1.3788 | 0.0001 |
| XM_417950.3 | 419814.0000 | 4047.0000 | 57.3618 | 21.9178 | 1.3880 | 0.0001 |
| XM_426419.4 | 428862.0000 | 2586.0000 | 2.4213 | 0.9211 | 1.3943 | 0.0130 |
| XM_003640625.2 | 428105.0000 | 713.0000 | 12.9918 | 4.9382 | 1.3956 | 0.0040 |
| NM_205268.1 | 396204.0000 | 1955.0000 | 9.3717 | 3.5546 | 1.3986 | 0.0001 |
| XM_004940550.1 | 101751235.0000 | 2993.0000 | 2.6576 | 1.0070 | 1.4000 | 0.0065 |
| NM_204464.1 | 395118.0000 | 1569.0000 | 4.3091 | 1.6317 | 1.4010 | 0.0003 |
| NM_001005431.1 | 421186.0000 | 1437.0000 | 131.5420 | 49.3750 | 1.4137 | 0.0001 |
| XM_422045.3 | 424199.0000 | 881.0000 | 1.1841 | 0.4352 | 1.4440 | 0.0153 |
| XM_003643398.2 | 430666.0000 | 3606.0000 | 0.9492 | 0.3463 | 1.4547 | 0.0001 |
| Novel001613.1 | Novel001613 | 5954.0000 | 2.8165 | 1.0186 | 1.4673 | 0.0002 |
| XM_415753.4 | 417504.0000 | 5516.0000 | 2.4901 | 0.8976 | 1.4721 | 0.0101 |
| XM_424041.4 | 396063.0000 | 873.0000 | 1.2192 | 0.4381 | 1.4765 | 0.0090 |
| NM_205522.1 | 396532.0000 | 770.0000 | 9.8477 | 3.3146 | 1.5710 | 0.0001 |
| XM_426829.4 | 429272.0000 | 1719.0000 | 1.5919 | 0.5228 | 1.6063 | 0.0001 |
| XM_424012.4 | 426356.0000 | 598.0000 | 3.3090 | 1.0844 | 1.6095 | 0.0015 |
| Novel002768.1 | Novel002768 | 1254.0000 | 0.8900 | 0.2892 | 1.6219 | 0.0113 |
| NM_001007477.3 | 396531.0000 | 753.0000 | 54.0512 | 17.5454 | 1.6232 | 0.0001 |
| NM_001277711.1 | 421697.0000 | 1163.0000 | 1.6251 | 0.5210 | 1.6411 | 0.0010 |
| NM_001167752.2 | 418056.0000 | 793.0000 | 21.4479 | 6.8143 | 1.6542 | 0.0001 |
| XM_004950438.1 | 101748338.0000 | 1044.0000 | 3.7069 | 1.1630 | 1.6724 | 0.0001 |
| XM_003643829.2 | 100857864.0000 | 1238.0000 | 1.6481 | 0.5122 | 1.6861 | 0.0006 |
| XM_416362.4 | 418130.0000 | 3766.0000 | 1.8400 | 0.5490 | 1.7449 | 0.0001 |
| XM_003643191.2 | 100858011.0000 | 621.0000 | 192.5700 | 56.7084 | 1.7638 | 0.0001 |
| XM_003641315.2 | 100859008.0000 | 2637.0000 | 0.4944 | 0.1420 | 1.7995 | 0.0013 |
| NM_001199544.1 | 423618.0000 | 3241.0000 | 0.8708 | 0.2494 | 1.8039 | 0.0001 |
| NM_205172.1 | 396084.0000 | 1296.0000 | 6.6936 | 1.9139 | 1.8063 | 0.0001 |
| NM_001199806.1 | 374016.0000 | 9643.0000 | 0.9611 | 0.2687 | 1.8386 | 0.0001 |
| NM_001271929.1 | 416874.0000 | 717.0000 | 5.7931 | 1.6095 | 1.8478 | 0.0001 |
| Novel000387.1 | Novel000387 | 744.0000 | 3.2896 | 0.9019 | 1.8668 | 0.0002 |
| NM_205489.1 | 396485.0000 | 601.0000 | 1136.3900 | 302.5300 | 1.9093 | 0.0001 |
| NM_001199704.1 | 423272.0000 | 1085.0000 | 3.0476 | 0.7881 | 1.9512 | 0.0001 |
| XM_004936544.1 | 374046.0000 | 5923.0000 | 7.2540 | 1.8645 | 1.9600 | 0.0002 |
| NM_204667.1 | 395387.0000 | 2452.0000 | 1.1617 | 0.2957 | 1.9743 | 0.0001 |
| XM_003643192.2 | 100858044.0000 | 546.0000 | 205.7770 | 51.7413 | 1.9917 | 0.0001 |
| Novel000349.1 | Novel000349 | 380.0000 | 14.8431 | 3.7312 | 1.9921 | 0.0001 |
| XM_004941552.1 | 428862.0000 | 3420.0000 | 2.9166 | 0.6957 | 2.0677 | 0.0010 |
| XM_004949790.1 | 101749670.0000 | 891.0000 | 2.8992 | 0.6840 | 2.0836 | 0.0002 |
| XM_004946094.1 | 427800.0000 | 2145.0000 | 4.7624 | 1.1169 | 2.0922 | 0.0001 |
| XM_424595.3 | 426991.0000 | 2775.0000 | 2.7309 | 0.6174 | 2.1451 | 0.0001 |
| XM_003643450.2 | 100858068.0000 | 990.0000 | 1.8752 | 0.4209 | 2.1555 | 0.0001 |
| NM_205009.2 | 395861.0000 | 1628.0000 | 1.7664 | 0.3258 | 2.4386 | 0.0001 |
| XM_426805.4 | 429249.0000 | 4248.0000 | 3.2934 | 0.5818 | 2.5009 | 0.0001 |
| XM_003642481.2 | 770495.0000 | 1870.0000 | 1.7116 | 0.2983 | 2.5206 | 0.0001 |
| NM_204976.1 | 395824.0000 | 2033.0000 | 2.1465 | 0.3609 | 2.5723 | 0.0001 |
| XM_004946241.1 | 427812.0000 | 3963.0000 | 0.7064 | 0.1119 | 2.6583 | 0.0001 |
| NM_204766.1 | 395534.0000 | 5979.0000 | 7.8329 | 1.1769 | 2.7346 | 0.0114 |
| NM_205471.1 | 396458.0000 | 2762.0000 | 6.5503 | 0.9600 | 2.7704 | 0.0001 |
| Novel001532.1 | Novel001532 | 6673.0000 | 1.8739 | 0.2605 | 2.8467 | 0.0001 |
| XM_424601.3 | 426999.0000 | 2170.0000 | 0.8265 | 0.0759 | 3.4443 | 0.0006 |
| XM_422727.4 | 424917.0000 | 1668.0000 | 1.1871 | 0.1034 | 3.5209 | 0.0001 |
| NM_001031489.1 | 428114.0000 | 558.0000 | 20.2802 | 1.3378 | 3.9221 | 0.0001 |
| NM_001001609.1 | 414341.0000 | 632.0000 | 35.9631 | 1.0742 | 5.0652 | 0.0001 |
| XM_429212.4 | 431660.0000 | 618.0000 | 20.8035 | 0.5664 | 5.1988 | 0.0001 |
| XM_425204.4 | 395402.0000 | 2200.0000 | 2.6513 | 0.0664 | 5.3192 | 0.0006 |
| ALDBGALT0000000470 | ALDBGALG0000000325 | 2176.0000 | 9.3184 | 6.0972 | 0.6119 | 0.0049 |
| ALDBGALT0000001233 | ALDBGALG0000000858 | 1045.0000 | 1.2189 | 0.2761 | 2.1422 | 0.0012 |
| ALDBGALT0000003468 | ALDBGALG0000002429 | 842.0000 | 1.0628 | 3.5964 | -1.7587 | 0.0022 |
| ALDBGALT0000003820 | ALDBGALG0000002664 | 881.0000 | 18.3248 | 12.2610 | 0.5797 | 0.0136 |
| ALDBGALT0000004545 | ALDBGALG0000003187 | 560.0000 | 49.5931 | 15.6450 | 1.6644 | 0.0001 |
| ALDBGALT0000005778 | ALDBGALG0000004022 | 850.0000 | 8.7926 | 16.0250 | -0.8660 | 0.0011 |
| ALDBGALT0000006415 | ALDBGALG0000004450 | 825.0000 | 0.0904 | 0.7902 | -3.1282 | 0.0094 |
| ALDBGALT0000006421 | ALDBGALG0000004455 | 4202.0000 | 3.9422 | 2.3726 | 0.7325 | 0.0018 |
| ALDBGALT0000007293 | ALDBGALG0000005049 | 789.0000 | 1.9215 | 5.4192 | -1.4959 | 0.0001 |
| ALDBGALT0000007306 | ALDBGALG0000005060 | 2026.0000 | 9.9112 | 2.6547 | 1.9005 | 0.0003 |
| ALDBGALT0000008084 | ALDBGALG0000005582 | 1974.0000 | 1.2412 | 0.4745 | 1.3874 | 0.0041 |
| ALDBGALT0000008086 | ALDBGALG0000005583 | 8725.0000 | 0.5965 | 0.1942 | 1.6191 | 0.0055 |
| ALDBGALT0000008096 | ALDBGALG0000005593 | 1582.0000 | 27.9717 | 18.6382 | 0.5857 | 0.0056 |
| TCONS_00155009 | XLOC_030020 | 334.0000 | 37.3137 | 67.8573 | -0.8628 | 0.0034 |
| TCONS_00363273 | XLOC_066477 | 449.0000 | 0.7061 | 0.8103 | -0.1986 | 0.0140 |
| TCONS_00994911 | XLOC_127296 | 8615.0000 | 0.5301 | 0.9221 | -0.7987 | 0.0082 |
| TCONS_01293759 | XLOC_387635 | 983.0000 | 0.2136 | 0.7075 | -1.7277 | 0.0133 |
| TCONS_02925749 | XLOC_179957 | 2012.0000 | 5.2888 | 2.9650 | 0.8349 | 0.0019 |
| TCONS_03027705 | XLOC_141626 | 13193.0000 | 0.7930 | 0.3477 | 1.1896 | 0.0022 |
| TCONS_03549595 | XLOC_108864 | 4000.0000 | 5.9491 | 8.5644 | -0.5257 | 0.0117 |
| TCONS_06324937 | XLOC_517981 | 435.0000 | 4.1025 | 1.8395 | 1.1572 | 0.0133 |
| TCONS_07170002 | XLOC_561688 | 2350.0000 | 0.6545 | 1.2679 | -0.9538 | 0.0086 |
| TCONS_09557654 | XLOC_809067 | 5368.0000 | 3.7957 | 1.0487 | 1.8558 | 0.0001 |
| TCONS_12040639 | XLOC_935007 | 2363.0000 | 1.1171 | 0.4965 | 1.1699 | 0.0027 |
| TCONS_13271494 | XLOC_933895 | 6521.0000 | 1.0478 | 0.3806 | 1.4611 | 0.0001 |
| TCONS_14646561 | XLOC_1194452 | 502.0000 | 3.3580 | 1.3606 | 1.3034 | 0.0073 |
| TCONS_14686939 | XLOC_1118038 | 1910.0000 | 0.2184 | 0.5889 | -1.4315 | 0.0137 |
| TCONS_15173814 | XLOC_1196171 | 4027.0000 | 62.8794 | 99.6140 | -0.6638 | 0.0131 |
| TCONS_15432353 | XLOC_1170550 | 1344.0000 | 0.8387 | 0.3648 | 1.2010 | 0.0137 |
| TCONS_15506568 | XLOC_1197139 | 11546.0000 | 1.6887 | 0.8029 | 1.0725 | 0.0001 |
| TCONS_16891537 | XLOC_1274507 | 4655.0000 | 1.4098 | 0.4581 | 1.6218 | 0.0001 |
| TCONS_17650206 | XLOC_1326840 | 363.0000 | 3.5681 | 7.3726 | -1.0470 | 0.0154 |
| TCONS_17738888 | XLOC_1317789 | 6984.0000 | 1.0957 | 0.5882 | 0.8976 | 0.0030 |
| TCONS_17763761 | XLOC_1317789 | 1750.0000 | 4.0634 | 1.2176 | 1.7387 | 0.0001 |
| TCONS_18443410 | XLOC_1362418 | 2477.0000 | 0.2134 | 0.7447 | -1.8033 | 0.0015 |
| TCONS_18636737 | XLOC_1366538 | 521.0000 | 2.3038 | 0.8447 | 1.4475 | 0.0132 |
| TCONS_18833676 | XLOC_1362418 | 3239.0000 | 0.1581 | 0.8765 | -2.4710 | 0.0001 |
