# Supplementary material for: Downregulated long noncoding RNA ALDBGALG0000005049 induces inflammation in chicken muscle suffered from selenium deficiency by regulating stearoyl-CoA desaturase
Source: Oncotarget. 2017 Apr 18;8(32):52761–74. doi: 10.18632/oncotarget.17187 (PMC5581067; doi:10.18632/oncotarget.17187)
Supplement: Supplementary file 3 [file oncotarget-08-52761-s003.doc]

**Supplementary Table 2: The potential targets of lncRNAs were predicted in cis**

| Gene_id (LncRNA) | Gene_id (mRNA) |
| --- | --- |
| ALDBGALG0000000325 | 418548 |
| ALDBGALG0000000325 | 418549 |
| ALDBGALG0000000325 | 418550 |
| ALDBGALG0000000325 | 425237 |
| ALDBGALG0000000325 | Novel000802 |
| ALDBGALG0000000325 | Novel000803 |
| ALDBGALG0000000325 | Novel000804 |
| ALDBGALG0000000858 | 418550 |
| ALDBGALG0000000858 | 418551 |
| ALDBGALG0000000858 | 418552 |
| ALDBGALG0000000858 | 418554 |
| ALDBGALG0000000858 | 771556 |
| ALDBGALG0000002429 | 395594 |
| ALDBGALG0000002664 | 420938 |
| ALDBGALG0000002664 | 420940 |
| ALDBGALG0000002664 | 420941 |
| ALDBGALG0000002664 | 770425 |
| ALDBGALG0000002664 | 770453 |
| ALDBGALG0000003187 | 100858163 |
| ALDBGALG0000003187 | 100858258 |
| ALDBGALG0000003187 | 100859094 |
| ALDBGALG0000003187 | 101747241 |
| ALDBGALG0000003187 | 101747315 |
| ALDBGALG0000003187 | 101747386 |
| ALDBGALG0000003187 | 101747463 |
| ALDBGALG0000003187 | 101747731 |
| ALDBGALG0000003187 | 101747796 |
| ALDBGALG0000003187 | 101747853 |
| ALDBGALG0000003187 | 101747961 |
| ALDBGALG0000003187 | 101748031 |
| ALDBGALG0000003187 | 101750786 |
| ALDBGALG0000003187 | 373951 |
| ALDBGALG0000003187 | 425709 |
| ALDBGALG0000003187 | 425968 |
| ALDBGALG0000003187 | 425969 |
| ALDBGALG0000003187 | 426356 |
| ALDBGALG0000003187 | 426357 |
| ALDBGALG0000003187 | 430570 |
| ALDBGALG0000003187 | 771890 |
| ALDBGALG0000003187 | Novel001755 |
| ALDBGALG0000003187 | Novel001758 |
| ALDBGALG0000004022 | 374242 |
| ALDBGALG0000004022 | 422341 |
| ALDBGALG0000004022 | 422342 |
| ALDBGALG0000004450 | 395131 |
| ALDBGALG0000004450 | 422804 |
| ALDBGALG0000004450 | 422805 |
| ALDBGALG0000004450 | 422806 |
| ALDBGALG0000004450 | 422807 |
| ALDBGALG0000004450 | 769392 |
| ALDBGALG0000004455 | 422820 |
| ALDBGALG0000004455 | Novel002202 |
| ALDBGALG0000005049 | 395706 |
| ALDBGALG0000005049 | 414746 |
| ALDBGALG0000005049 | 423763 |
| ALDBGALG0000005049 | 423764 |
| ALDBGALG0000005049 | 423767 |
| ALDBGALG0000005049 | 423768 |
| ALDBGALG0000005049 | 428952 |
| ALDBGALG0000005049 | 428953 |
| ALDBGALG0000005049 | 428954 |
| ALDBGALG0000005049 | Novel002449 |
| ALDBGALG0000005049 | Novel002455 |
| ALDBGALG0000005060 | 423796 |
| ALDBGALG0000005060 | 425328 |
| ALDBGALG0000005060 | 427031 |
| ALDBGALG0000005583 | 424882 |
| ALDBGALG0000005583 | 424883 |
| ALDBGALG0000005593 | 100857766 |
| ALDBGALG0000005593 | 101751236 |
| ALDBGALG0000005593 | 101752124 |
| ALDBGALG0000005593 | 101752296 |
| ALDBGALG0000005593 | 396191 |
| ALDBGALG0000005593 | 407088 |
| ALDBGALG0000005593 | 424913 |
| ALDBGALG0000005593 | 424914 |
| ALDBGALG0000005593 | 424915 |
| ALDBGALG0000005593 | 424916 |
| ALDBGALG0000005593 | 424917 |
| ALDBGALG0000005593 | 424918 |
| ALDBGALG0000005593 | 424919 |
| ALDBGALG0000005593 | 424920 |
| ALDBGALG0000005593 | 424921 |
| ALDBGALG0000005593 | 431014 |
| XLOC_066477 | Novel000523 |
| XLOC_066477 | Novel000524 |
| XLOC_108864 | 418090 |
| XLOC_108864 | 418091 |
| XLOC_108864 | 418092 |
| XLOC_108864 | 418093 |
| XLOC_1118038 | Novel002159 |
| XLOC_1118038 | Novel002165 |
| XLOC_1118038 | Novel002166 |
| XLOC_1118038 | Novel002167 |
| XLOC_1129433 | Novel002187 |
| XLOC_1170550 | 404779 |
| XLOC_1170550 | 422439 |
| XLOC_1170550 | 428731 |
| XLOC_1194452 | 408035 |
| XLOC_1194452 | 422696 |
| XLOC_1194452 | 428772 |
| XLOC_1194452 | 771805 |
| XLOC_1196171 | 395113 |
| XLOC_1196171 | 395979 |
| XLOC_1196171 | 422704 |
| XLOC_1196171 | 422705 |
| XLOC_1196171 | 422708 |
| XLOC_1196171 | 422709 |
| XLOC_1196171 | 425210 |
| XLOC_1196171 | 425211 |
| XLOC_1196171 | 426361 |
| XLOC_1196171 | 426362 |
| XLOC_1196171 | 426710 |
| XLOC_1196171 | 771920 |
| XLOC_1196171 | 772145 |
| XLOC_1196171 | 772178 |
| XLOC_1196171 | Novel002162 |
| XLOC_1196171 | Novel002226 |
| XLOC_1196171 | Novel002279 |
| XLOC_1197139 | 395168 |
| XLOC_1197139 | 396319 |
| XLOC_127296 | 101749410 |
| XLOC_127296 | 396402 |
| XLOC_1274507 | 428862 |
| XLOC_1317789 | 374253 |
| XLOC_1317789 | 423796 |
| XLOC_1317789 | 425328 |
| XLOC_1317789 | 427031 |
| XLOC_1317789 | 768380 |
| XLOC_1326840 | 100857412 |
| XLOC_1326840 | 423976 |
| XLOC_1362418 | 395727 |
| XLOC_1362418 | 396415 |
| XLOC_1362418 | 424141 |
| XLOC_1362418 | 424142 |
| XLOC_1362418 | 429021 |
| XLOC_1362418 | 771375 |
| XLOC_1362418 | Novel002578 |
| XLOC_1366538 | 424230 |
| XLOC_1366538 | 424231 |
| XLOC_1366538 | Novel002580 |
| XLOC_1366538 | Novel002602 |
| XLOC_141626 | 418486 |
| XLOC_141626 | 418487 |
| XLOC_141626 | 418488 |
| XLOC_141626 | 418489 |
| XLOC_387635 | 396338 |
| XLOC_387635 | 419007 |
| XLOC_517981 | 416553 |
| XLOC_517981 | 416554 |
| XLOC_517981 | 416555 |
| XLOC_517981 | 416556 |
| XLOC_517981 | 416558 |
| XLOC_517981 | 416559 |
| XLOC_517981 | 416560 |
| XLOC_517981 | 416561 |
| XLOC_517981 | 427671 |
| XLOC_517981 | 770994 |
| XLOC_517981 | Novel001211 |
| XLOC_561688 | 417332 |
| XLOC_561688 | 417333 |
| XLOC_561688 | Novel001322 |
| XLOC_809067 | 378788 |
| XLOC_809067 | 420254 |
| XLOC_809067 | 420255 |
| XLOC_809067 | 420257 |
| XLOC_809067 | 420259 |
| XLOC_809067 | 420261 |
| XLOC_809067 | Novel001422 |
| XLOC_809067 | Novel001519 |
| XLOC_809067 | Novel001612 |
| XLOC_809067 | Novel001613 |
| XLOC_809067 | Novel001653 |
| XLOC_809067 | Novel001654 |
| XLOC_933895 | 421692 |
| XLOC_933895 | Novel001989 |
| XLOC_935007 | 374135 |
| XLOC_935007 | 426929 |
